# Spatiotemporal patterns of influenza in Western Australia

**DOI:** 10.1016/j.puhip.2025.100602

**Published:** 2025-03-15

**Authors:** Kefyalew Addis Alene, Hannah C. Moore, Archie C.A. Clements, Beth Gilmour, Dylan D. Barth, Rebecca Pavlos, Ben Scalley, Christopher C. Blyth

**Affiliations:** a School of Population Health, Faculty of Health Sciences, Curtin University, Perth, Western Australia, Australia; b Wesfarmers Centre of Vaccines and Infectious Diseases, The Kids Research Institute Australia, Perth, Western Australia, Australia; c Geospatial and Tuberculosis Research Team, The Kids Research Institute Australia, Perth, Western Australia, Australia; d Queen’s University Belfast, Belfast, UK; e Western Australian Department of Health, Perth, WA, Australia; f North Metropolitan Health Service, Perth, WA, Australia; g School of Medicine, The University of Western Australia, Perth, WA, Australia; h Department of Infectious Diseases, Perth Children's Hospital, Perth, WA, Australia; i PathWest Laboratory Medicine, QEII Medical Centre, Nedlands, Perth, WA, Australia

**Keywords:** Epidemiology, Spatial, Spatiotemporal, Influenza, Western Australia

## Abstract

**Background:**

Understanding the geospatial distribution of influenza infection and the risk factors associated with infection clustering can inform targeted preventive interventions. We conducted a geospatial analysis to investigate the spatial patterns and identify drivers of medically attended influenza infection across all age groups in Western Australia (WA).

**Methods:**

Data for confirmed influenza cases were obtained from the WA Notifiable Infectious Diseases Database for the period 2017–2020. Data were also obtained for vaccination coverage, meteorological parameters, socioeconomic indicators, and healthcare access. Spatial clustering of influenza incidence was identified using Global Moran's I and Getis-Ord statistic. Bayesian spatial models were used to identify factors associated with spatial clustering of infection.

**Results:**

Of the 36,228 influenza cases reported, over half (18,773, 51·8 %) were in individuals aged between 15 and 64 years and more than three quarters (28,545, 78·9 %) in the Perth metropolitan region. The annual incidence rate ranged from 2·7 per 1000 population in individuals aged between 15 and 64 years to 5·2 per 1000 population in children <5 years of age. For all age groups, the lowest incidence (0·4 per 1000 population) and the highest incidence rate (8·8 per 1000 population) were reported during and pre-the COVID-19 pandemic respectively. The influenza incidence rate shows both seasonal and spatial variation. Spatial clustering was significantly associated with distance to the nearest health facility in minutes (*B* = −0·181; 95 %CrI: 0·279, −0·088) and annual mean temperature in degrees Celsius (*B* = 0·171; 95 %CrI: 0·015, 0·319).

**Conclusions:**

Spatial clustering of influenza incidence was significantly associated with climatic conditions and healthcare access.

## 1 Introduction

Seasonal influenza virus infection causes substantial morbidity and mortality every year, particularly in older adults and children <5 years of age [[1], [2], [3], [4]]. A recent study has estimated that influenza causes 32·1 million cases of illness and 5·6 million hospital admissions each year globally among adults [5]. It has also been estimated that influenza causes approximately 389,000 deaths globally, with 34,800 deaths occurring in children <5 years of age in 2018 [6,7]. In Australia, prior to circulation of the SARS-CoV-2 virus, influenza was a major health problem causing significant mortality (2·6 per 100,000 population) and hospital admissions (7·4 per 100,000 population), with the highest mortality and morbidity rates reported among adults aged 75 years or above and children <5 years [8]. In 2019, Australia experienced the highest number of influenza infections ever, with over 300,000 laboratory-confirmed cases [9].

Influenza infection rates are highly seasonal and differ significantly between geographic regions across the globe [10]. In temperate regions, annual epidemics of influenza mainly occur during the cold winter season [11]. Timing of these annual influenza epidemics varies at national and local levels and from year to year. Variations in timing can be partially explained by the impacts of climatic and environmental factors on the transmission of the virus [12]. Previous studies have reported that climatic factors including rainfall, humidity and temperature play a key role in the transmission of influenza in the community [[13], [14], [15]]. Ecological-level factors such as socio-economic index and population density can also influence the spatiotemporal distribution of influenza [13]. Several other risk factors such as vaccination coverage and distance to health facility might also contribute to the spatial variation and seasonal patterns of influenza infection [16].

Knowledge of the relationship between influenza infection, vaccination coverage, demographic and climatic factors in smaller geographical areas might be useful for planning targeted vaccination programs, allowing policy makers and public health practitioners to predict hotspot areas and timing of influenza peak activity to optimise delivery of vaccines. Therefore, the aim of this study was to investigate the spatial patterns of medically attended influenza infections and relationships with area-level vaccination coverage and meteorological parameters in Western Australia (WA).

## 2 Methods

### 2.1 Study area

Western Australia (WA) is the largest state in Australia by area, with a total area of 2·5 million square kilometres. According to the most recent census report, the population of WA was 2.7 million people in 2022 [17]. Climate conditions in WA are very diverse from the tropical north, to the central desert regions and the temperate southwest coastal areas [18]. The climate in the southwest experiences four distinct seasons with hot and dry summers (December–February) and mild and rainy winters (June–August). The climate in the tropical north is characterised by a wet season and a dry season (April–November), while the climate in the central desert regions is arid [18]. Average annual rainfall varies across the state and decreases away from the coast. In WA, the median weekly income was $848 for individuals and $2214 for families in 2021 [17].

### 2.2 Study design

This study was conducted using an ecological study design where correlations between medically attended influenza infection rates and exposures variables were measured at postcode level.

### 2.3 Data sources

We used different data sources for the primary outcome and exposure variables. Our primary outcome measure was the number of reported influenza infections. A confirmed case of influenza was defined on the basis of laboratory confirmation by culture, nucleic acid testing (NAT), antigen assay testing, or antibody testing. Confirmed cases of influenza were obtained from the WA Notifiable Infectious Diseases Database (WANIDD) and data extracted were deidentified and presented as monthly aggregated counts at postcode level for the period between 2017 and 2020 across all age groups.

Population data (i.e., number of people in each postcode) were obtained from the Australian Bureau of Statistics. Population density for each postcode was calculated by dividing the total number of people in each postcode by the land area of the postcode to obtain the number of people per square kilometre. The data sources of the covariates with their definitions are provided in the supplementary information (Table S1).

Our exposures of interests were vaccination coverage, meteorological parameters, socioeconomic indicators, and access to healthcare. These variables were selected based on evidence of association with influenza infection from previous studies and based on the availability of state-wide representative data. Influenza vaccination coverage was determined by WA Health using the number of individuals with a recorded influenza vaccine dose between April and September 2019 registered in the Australian Immunisation Register. Vaccination coverage data were aggregated by age groups and standard health regions which include seven rural and remote regions (Kimberley, Pilbara, Midwest, Goldfields, Wheatbelt, Southwest, Great Southern) and three metropolitan regions (North Metropolitan, South Metropolitan, and East Metropolitan). Socioeconomic status (SES) data were obtained from the Australian Bureau of Statistics. These data were derived from the Socio-Economic Index for Area (SEIFA) codes [19], specifically the index of relative socio-economic disadvantage decile, index of relative socio-economic advantage and disadvantage decile, index of economic resources decile, and index of education and occupation decile. Monthly aggregated meteorological parameters such as temperature, precipitation, water vapor pressure, solar radiation, and wind speed were obtained from the WorldClim database [20]. In addition, data on healthcare access (i.e., travel minutes to the nearest general practitioner (GP) or hospital) were obtained from the Malaria Atlas Project (MAP) [21].

A polygon shapefile for WA postcodes was obtained from the Database for Global Administrative Areas (GADM), a free online database [22]. The dependant variables (influenza cases) were geo-referenced using the patients’ residential postcodes, and covariates were linked to the dependant variable using ArcGIS (ESRI, Redlands, CA) geographical information system (GIS) software.

### 2.4 Data analysis

**Influenza incidence rate**: As a descriptive analysis, influenza incidence rate was calculated for different age groups (<5 years, 5–64 years and ≥65 years) by each year. Incidence rates were calculated by dividing the total number of new influenza cases, by the population size of the same age group in the corresponding postcode, multiplied by 1000 to obtain an incidence rate per 1000 population.

**Standardized incidence ratio:** Age and year adjusted standardized incidence ratios (SIR) were calculated. For each postcode i, i = 1 … n, the SIR was calculated as the ratio of the observed number of influenza cases in the postcode ($Y_{i}$) to the expected number of influenza cases ($E_{i}$) in the postcode across the study period:

$${SIR}_{i}=Y_{i}\;/E_{i}$$

The expected count $E_{i}$ represented the total number of influenza cases that one would expect if the population of postcode i had the same risk as the state population. The expected number of influenza cases for each postcode ($E_{i}$) was computed as:

$$E_{i}=r_{j}^{\left(s\right)}n_{j}^{\left(s\right)}\text{,}$$

Where $r_{j}^{\left(s\right)}$ is the overall crude influenza incidence rate for WA (i.e., total number of influenzas cases divided by total population in all postcodes), and $n_{j}^{\left(s\right)}$ is the population of each postcode i.

**Spatial autocorrelation analysis:** The global Moran's I and the Getis-Ord statistic were used to identify clusters of high influenza incidence at postcode level across all regions of WA. The global Moran's I statistic was used to assess the presence, strength, and direction of spatial autocorrelation over the state of WA and to test the assumption of spatial independence in implementing the spatial pattern analysis. The Getis-Ord statistic was used to detect local clustering of medically attended influenza infection. Maps produced from the Moran's I statistics and the Getis-Ord statistics show the existence of medically attended influenza infection clusters and identify the locations of potential hotspot areas.

**Bayesian spatial analysis:** Although SIRs can be useful in some settings, in postcodes with small populations the expected counts will be very low, and SIRs may be insufficiently reliable for reporting. Therefore, we also estimated influenza infection risk by using Bayesian spatial models that enable estimates to borrow information from neighbouring postcodes and incorporate information from covariates, resulting in the smoothing or shrinking of extreme values in areas with small populations.

The model was constructed using covariates, unstructured random effects, and spatially structured random effects. The observed numbers of influenza cases $Y_{i}$ in postcode i, were modelled using a Poisson distribution with mean $E_{i}\theta_{i}$, where $E_{i}$ is the expected number of influenzas cases and $\theta_{i}$ is the relative risk in postcode i. The logarithm of the relative risk $\theta_{i}$ was expressed as the sum of an intercept that models the overall risk level of influenza, a vector of covariates and their coefficients, and random effects to account for extra-Poisson variability. The model for the spatial data is expressed as follows:

$$Y_{i}\;∼\;P_{o}\left(E_{i}\theta_{i}\right),i=1,\ldots ,n$$

$$\log\left(\theta_{i}\right)={a+d_{i}B+u}_{i}+v_{i}$$

Here, a represents the overall risk of influenza in the state, $d_{i}B$ represents the covariates that were included in the model to quantify the source of variability. Where $d_{i}$ = (1, $d_{i1},\ldots ,d_{ip})$ is the vector of p covariates corresponding to postcode i and $B=(B_{0},B_{1},\ldots ,B_{p}$) is the coefficient vector of the covariates. In this setting, for a one-unit increase in covariate ${\;d}_{j\;},j=1,\ldots ,p$, the relative risk increases by a factor of $\exp\left(B_{j}\right)$, holding all other covariates constant. Additionally, $u_{i}$ is a random effect specific to postcode i to model spatial dependence between the relative risks, and $v_{i}$ is an unstructured exchangeable component that models uncorrelated noise, $v_{i}\;∼\;N(0,\sigma_{v}^{2}$).

The spatial random effect $u_{i}$ was assigned a Conditional Autoregressive (CAR) distribution using a queen neighbourhood weight matrix, which specified that two areas are neighbours if they share a common boundary. Specifically,

$${u_{i}/u}_{\_\_i}\;∼\;N\left({ŭ}_{\sigma i},\frac{\sigma_{u}^{2}}{n_{\sigma i}}\right)$$

Where ${ŭ}_{\sigma i}=n_{\sigma i}^{-1}\sum_{j\in \sigma i}u_{j},\sigma i$ and $n_{\sigma i}$ represent, respectively, the set of neighbours and the number of neighbours of postcode i. The unstructured component $v_{i}$ was modelled as independent and identically distributed normal variables with zero mean and variance $\sigma_{v}^{2}$.

Before fitting the model, all covariates were checked for multi-collinearity using Pearson correlation coefficients (Figs. S1 and S2) and variance inflation factors (VIF). Those variables with a VIF greater than six were excluded from the final model. Vaccination coverage, socioeconomic index (i.e., index of relative socio-economic advantage and disadvantage decile), distance to health facility (i.e., hospital or GP), monthly mean temperature, and monthly mean precipitation were eligible covariates to be included in the final model. Since these independent variables had different units and scales of measurement that would have unknown threshold effects, the variables were normalised using their mean and standard deviation ([X-mean]/SD). This method also helped with identifiability in the estimation of the posterior distribution of the coefficients. All the analyses were conducted using R and ArcGIS software.

## 3 Results

### 3.1 Demographic characteristics of influenza

In total, 36,228 influenza cases were reported to WANIDD between January 2017 and December 2020. Of these influenza cases, 18,773 (51·8 %) were in individuals aged between 15 and 64 years and 28,545 (78·9 %) were from the Perth metropolitan region. The largest number of cases was reported in 2019, which accounted for 64·0 % (n = 23,198) of the cases over the study period. The average number of monthly influenza cases in WA for all age groups before the COVID-19 pandemic period was 973 (ranging from 102 cases in April 2017 to 10,514 cases in June 2019). Geographic locations were unknown for 72 influenza cases; thus, we excluded them from the spatial analysis. Table 1 summarises the demographic characteristics of influenza cases in WA.

**Table 1:** Number and percentage of influenza cases reported in Western Australia stratified by age groups, years, and regions, 2017–2020.

Characteristics	Total number of influenza cases n	Percentage from the total cases %
**Ages**		
<6 months	204	0.6
6 months–4 years	3410	9.4
5–14 years	7503	20.7
15–64 years	18773	51.8
≥65 years	6338	17.5

**Years**		
2017	5994	16.5
2018	5839	16.1
2019	23198	64.0
2020	1197	3.3

**Regions**^a^		
Goldfields	696	1.9
Great Southern	814	2.3
Kimberley	822	2.3
Perth Metropolitan	28545	78.9
Midwest	1447	4.0
Pilbara	821	2.3
Southwest	2224	6.2
Wheatbelt	787	2.2

a Regions were unknown for 72 cases.

### 3.2 Influenza incidence rate

The overall influenza incidence rate across the study period was 3·5 cases per 1000 population. The annual influenza incidence rate was 2.7 per 1000 population among individuals aged between 15 and 64 years, compared with 5.2 per 1000 among those under 5 years. The incidence rate of influenza also varied by year, with the lowest incidence rate (0·4 per 1000 population) reported in 2020 (i.e., during the COVID-19 pandemic) and the highest incidence rate (8·8 per 1000 population) reported in 2019 (Table 2).

**Table 2:** Annual incidence rate of influenza (per 1000 population) in Western Australia, stratified by age groups, 2017–2020.

Year	<5 years		5–14 years		15–64 years		≥65 years		Total	
	Cases	Incidence	Cases	Incidence	Cases	Incidence	Cases	Incidence	Cases	Incidence
2017	477	2.7	802	2.4	3297	1.9	1401	3.9	5977	2.3
2018	633	3.7	987	3.0	3297	1.9	906	2.4	5823	2.2
2019	2406	14.0	5580	16.5	11389	6.6	3789	9.7	23164	8.8
2020	94	0.5	126	0.4	746	0.4	226	0.6	1192	0.4
**Total**	3610	5.2	7495	5.6	18729	2.7	6322	4.1	36156	3.5

**Fig. 1:**
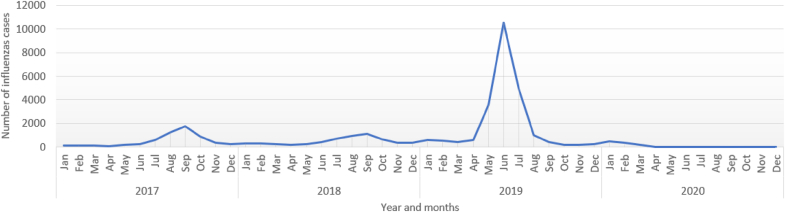
The monthly reported number of influenzas cases in Western Australia, 2017–2020.

**Fig. 2:**
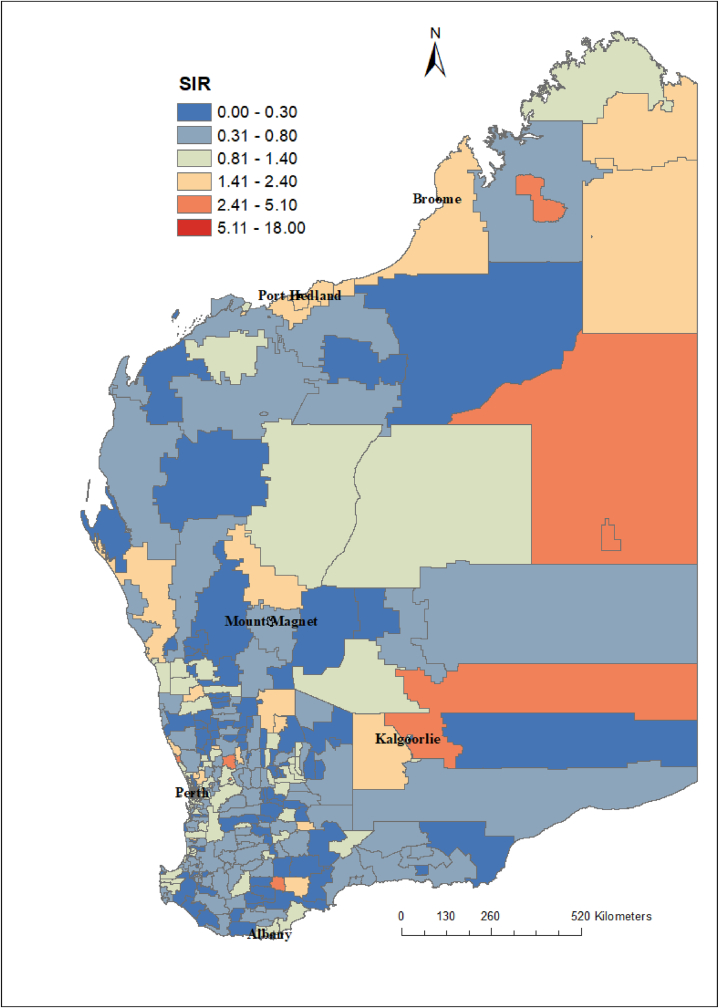
Age and year standardized incidence rate (SIR) of influenza cases at postcode level in Western Australia, 2017–2020. In postcode with SIR around one (colour yellow) the number of influenza cases observed is the same as the number of expected cases. In postcode SIR >1 (colour red), the number of influenza cases observed is higher than the expected cases. Postcodes where SIR <1 (colour blue and green) have fewer influenza cases observed than expected.

**Fig. 3:**
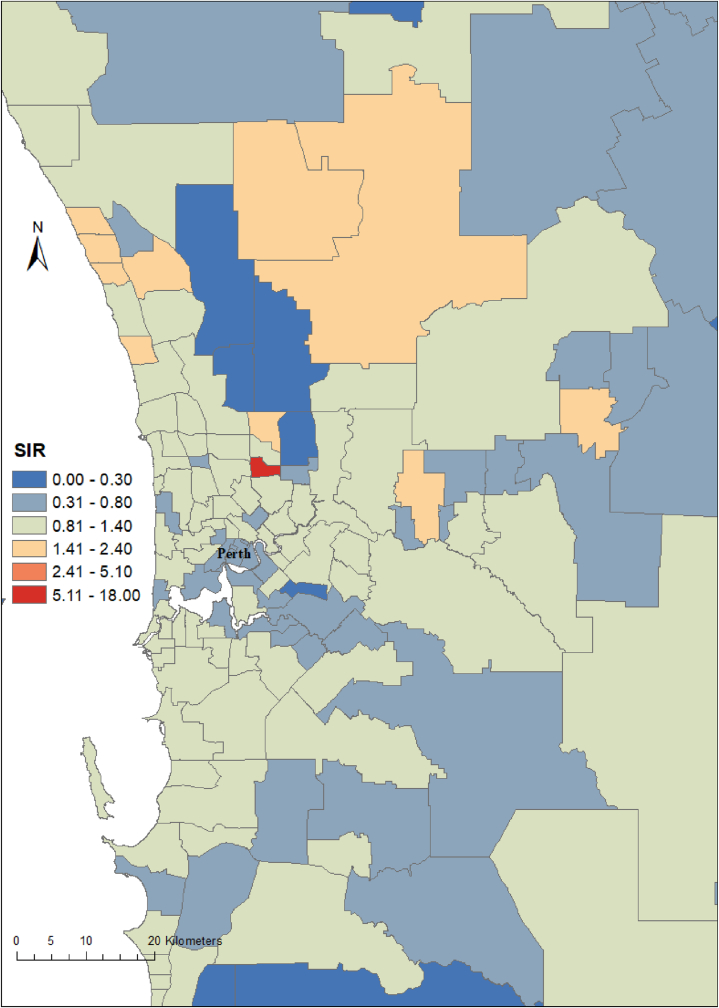
Age and year standardized incidence rate (SIR) of influenza cases at postcode level for Perth metropolitan areas in Western Australia, 2017–2020.

Influenza cases displayed seasonal variations with the highest number of cases reported between June and October in 2017 and 2018 and between April and August in 2019 (Fig. 1). Influenza incidence varied substantially at the postcode level, with SIRs ranging from 0 to 18 per 1000 population. The SIR of influenza cases at postcode level is presented in Fig. 2, Fig. 3.

### 3.3 Spatial clustering of influenza

**Fig. 4:**
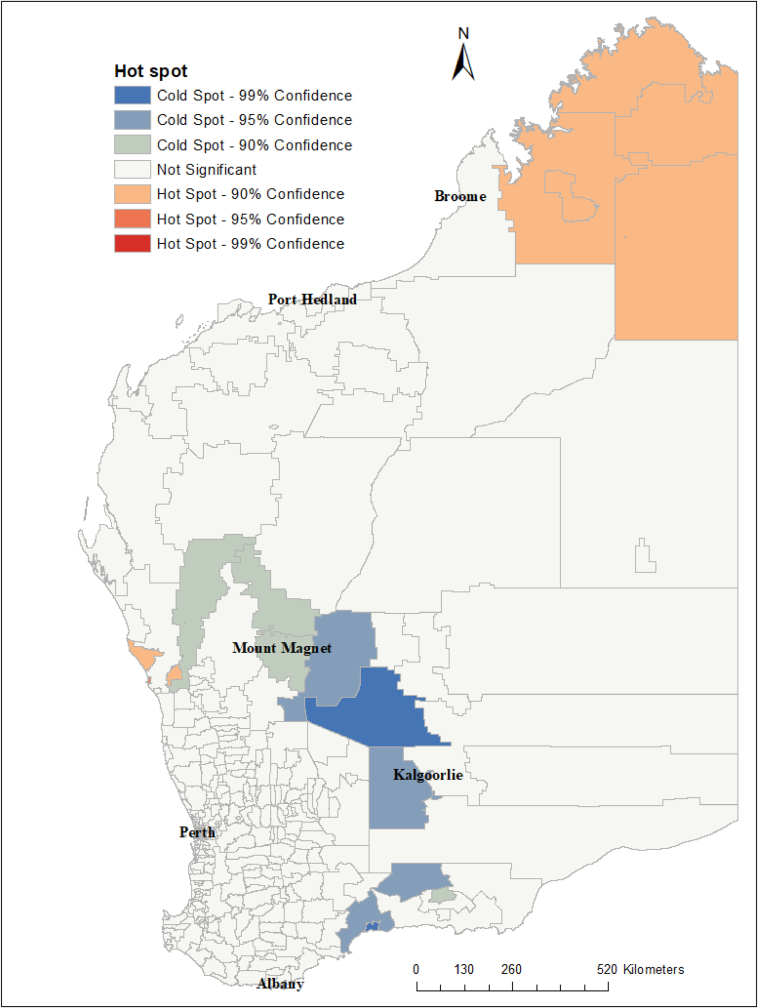
Spatial clustering of influenza incidence in Western Australia based on the Getis-Ord Gi∗ statistics, 2017–2020. There were no statistically significant relationships for many of the postcodes.

**Fig. 5:**
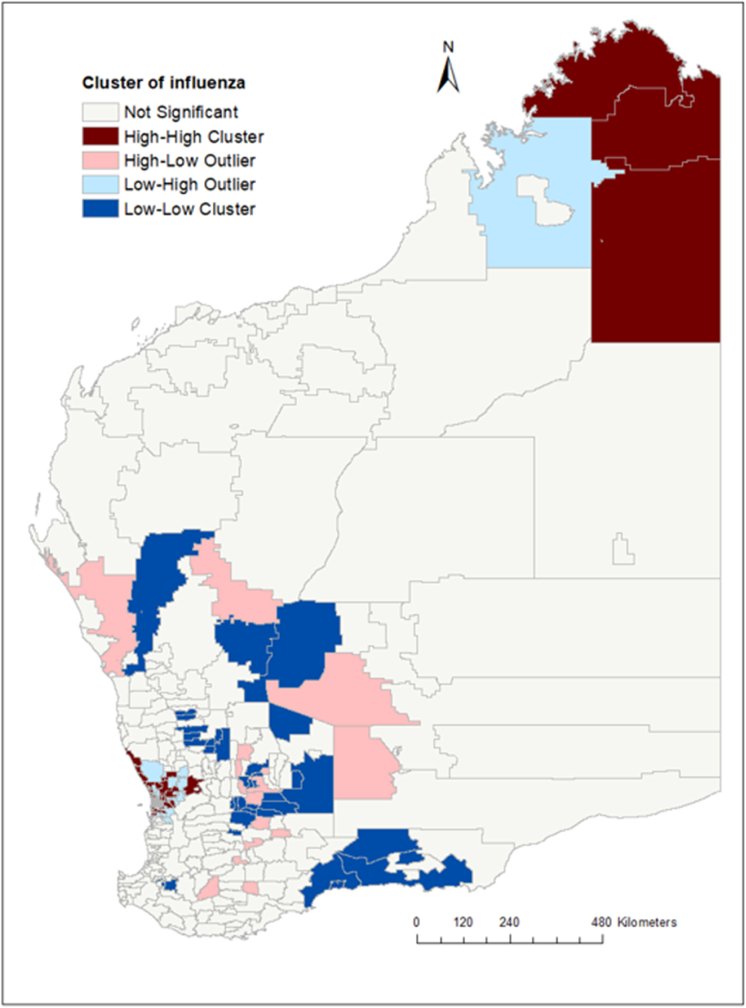
Spatial clustering of influenza incidence in Western Australia based on local Moran's I statistics, 2017–2020.

The global Moran's index statistic for influenza incidence was 0.03 (p-value <0.001), indicating the presence of significant, positive spatial autocorrelation in influenza incidence rate over the whole study area. Based on our clustering analyses using the Getis–Ord G statistic, some postcodes were identified as hot spots and cold spots (Fig. 4). The hot spot postcodes, indicating higher than expected incidence of influenza compared to the state average, were in the northern part of WA in the Kimberley region and in the Perth metro region (Fig. 5), while the cold spot postcodes were located in the Goldfields and Midwest regions. In the local Moran's I analysis, postcodes located in metropolitan regions and northern part of WA (the Kimberley region) showed a high-high type of relationship, meaning that these postcodes had a high incidence of influenza cases and the surrounding postcodes also had high influenza incidence (Fig. 5). Some postcodes in the metro areas had a high-low type of relationship which indicated that there was a high incidence of influenza in these postcodes, surrounded by postcodes with a low incidence of influenza. Low-low clusters of influenza were found in the Goldfield and Midwest regions (Fig. 5).

### 3.4 Influenza vaccination coverage

**Fig. 6:**
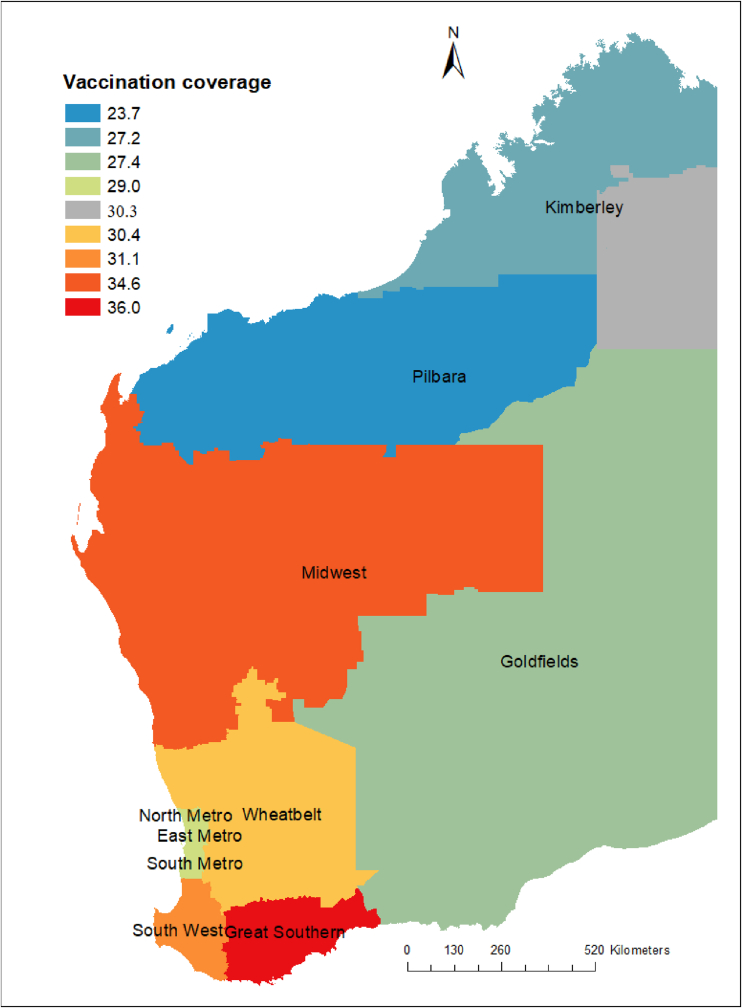
2019 Vaccination coverage in Western Australia at regional level.

**Fig. 7:**
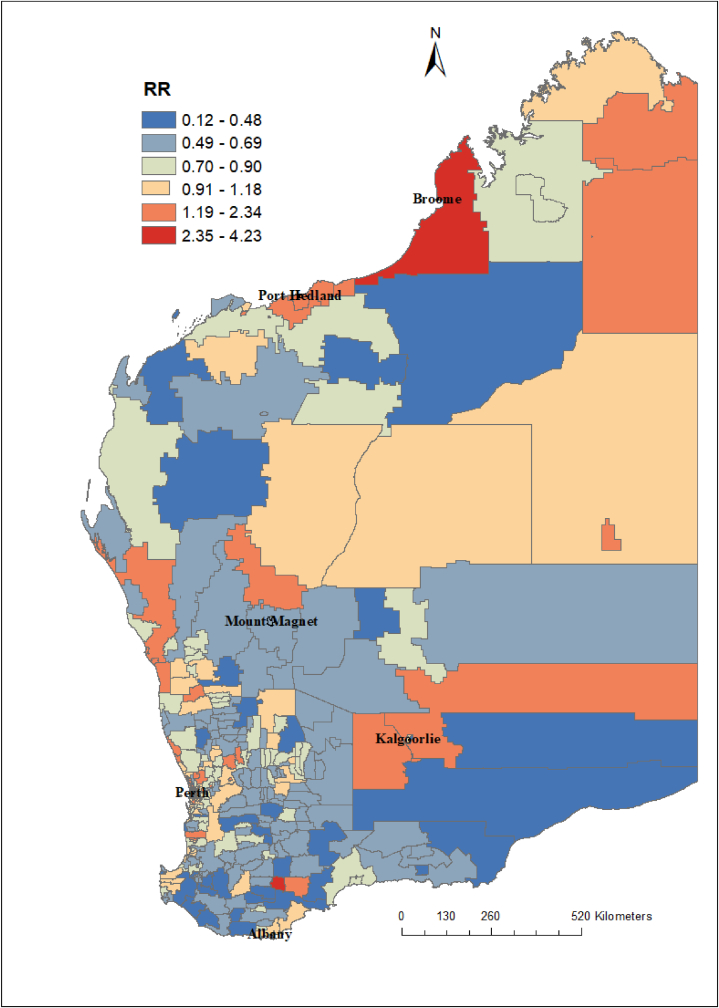
Relative risk (RR) of influenza infection at postcode level in Western Australia based on the posterior spatially structured random effects, 2017–2020. The relative risk (RR) quantified whether a postcode has higher (RR > 1) or lower (RR < 1) risk than the average risk in the state population. For example, if (RR = 2), this means that the risk of the postcode is two times the average risk in the state population.

Influenza vaccination coverage data were available at regional level only for 2019 (Fig. 6). These data indicated that the influenza vaccination coverage (i.e., the proportion of people vaccinated against influenza virus) was 51·3 % for children age between 6 months and 5 years and 61·6 % for individuals aged ≥65 years (Table 3) in 2019. The geographical distribution of covariates included in the models such as socioeconomic index (Fig. S3), distance to health facility (Fig. S4), annual mean temperature (Fig. S5), annual mean precipitation (Fig. S6), and population density (Fig. S7) are presented in the supplementary files. To see spatial changes by age group and year, we have also presented maps of subgroup analysis in Fig. 7 and supplementary information (Figure S8–15).

**Table 3:** Seasonal influenza vaccination coverage (% of population) for all doses in Western Australia stratified by age groups and regions in 2019.

Regions	6 months to <5 years	5 to <15 years	15 to <65 years	65+ years
Goldfields	54.5	28.2	20.9	50.8
Great Southern	50.1	28.7	26.6	66.6
Kimberley	51.0	30.0	23.5	32.8
Mid-west	59.5	33.9	26.3	60.9
Pilbara	51.6	25.3	19.3	31.0
Southwest	43.2	26.0	22.6	62.8
Wheatbelt	55.1	26.1	21.6	54.1
Metropolitan areas^a^	51.6	28.5	19.7	62.3
**Total**	51.3	28.3	20.3	61.6

a North metro, East metro, and south metro.

### 3.5 Ecological-level factors associated with spatial clustering of influenza

Table 4 shows the Bayesian multivariable Poisson regression model of ecological-level factors associated with incidence of influenza in WA. The model showed two factors significantly associated with the incidence of influenza cases: distance travelled to the nearest health facility in minutes (*B* = −0·181; 95 %CrI: 0·279, −0·088) was negatively associated, and the annual mean temperature per degree Celsius (*B* = 0·171; 95 %CrI: 0·015, 0·319) was positively associated with influenza incidence. Other ecological level factors included in the models such as vaccination coverage, socioeconomic index, and annual mean precipitation were not significantly associated with influenza incidence in WA. After accounting for the ecological-level factors in the model, the posterior mean of spatially structured random effects was found to be clustered in the state (Fig. S8). This indicates that a substantial amount of postcode level heterogeneity in influenza remained unexplained by the ecological level factors included in our models.

**Table 4:** Regression coefficient mean and 95 % credible intervals (CrI) of covariates included in a Bayesian spatial model.

Covariates	Regression coefficients Mean	Regression coefficient 95 % CrI
Vaccination coverage (percentage)	0.097	−0.003, 0.203
Socioeconomic index (decile)	−0.054	−0.129, 0.019
**Walking distance to the nearest health facility (minutes)**	**−0.181**	**−0.279, -0.088**
**Annual mean temperature (°C)**	**0.171**	**0.015, 0.319**
Annual mean precipitation (mm)	0.060	−0.062, 0.181
Population density (person per secure kilometres)	0.016	−0.078, 0.109
Intercept	−0.314	−0.379, −0.253

CrI: credible interval; bold fonts show ‘statistically significant’ results within a Bayesian framework (no zero within the 95 % CrI).

## 4 Discussion

This study investigated the spatiotemporal distribution of medically attended influenza virus infection in WA for the period between 2017 and 2020. Significant spatial clustering of influenza cases was observed and associated with ecological level factors such as distance travelled to health facility and annual mean temperature. Areas with high influenza incidence can be prioritized for educational campaigns and deployment of primary prevention strategies including those seeking to increase vaccination coverage.

Seasonal patterns of influenza are determined by multiple factors including environmental, virological, demographic and climatic factors as well as public health interventions [23]. In our study, influenza cases have shown seasonal variations and varied by year. The lowest incidence rate of influenza was reported in 2020 during the COVID-19 pandemic and the highest incidence rate was reported in 2019. The highest number of influenza cases reported in 2019 could be due to an unusually early and prolonged season dominated by A(H3N2) viruses and a larger susceptible population following a mild 2018 season. The low number of cases reported during the COVID-19 pandemic in 2020 has been attributed to public health measures taken to stop the spread of COVID-19 in the community [24]. Various preventive measures have been taken at a global, regional, and national level to reduce the risk of COVID-19 transmission [25]. In WA, this included strict measures which included the closing of borders to prevent importing cases, closing schools and public facilities (early in 2020), the lock-down of social and economic activities, encouraging social distancing, and wearing personal protective equipment. All these measures could also have had a positive impact in the prevention and control of influenza infection [24,26].

Another important finding of our study was that the incidence of influenza was negatively associated with travel time to the nearest health facilities (such as hospitals or GPs). This means that for people who lived in postcodes that were further away from healthcare facilities, the lower the influenza incidence would be. This finding maybe explained by the under reporting of influenza cases in remote regions, who often operate in resource-limited environments. In addition, healthcare access is also limitation in remote regions which may have an impact on the low ascertainment of influenza cases. Access to healthcare is an important consideration and despite having one of the best health-care services in the world, Australia's vast geographical environment creates inequalities in access to healthcare for some population groups [27]. Alternatively, as has been demonstrated by other pathogens including *Clostridium difficile* [28], healthcare facilities might be a source of influenza infection for the catchment population and those who live in postcodes near to healthcare facilities. Further investigation is required to determine why postcodes nearer to healthcare facilities are at a higher risk of influenza infection in WA.

This study showed annual mean temperature to be significantly associated with influenza incidence. The mechanisms of this relationship are not understood, in temperate regions of the Northern and Southern hemispheres influenza incidence is higher in winter months [29], but epidemics in tropical and subtropical regions are more diverse and often occur during periods of high temperature and humidity [30]. Modelling studies suggest that these diverse epidemic patterns may relate to temperature differentials and that infection risk is highest when there is a large mean temperature differential in relatively warm climate [31].

There were some limitations to this study that need to be acknowledged. First, our study did not include potential cofounding variables such as household size, ethnicity, and behavioural factors such as smoking that might better explain spatial variation, as these variables were not available in our dataset. Second, the data used from the WANIDD does not contain specific information on hospitalization status for influenza cases. As such, we were unable to stratify cases into hospitalized versus non-hospitalized groups, which may have implications for understanding the relationship between distance to healthcare facilities and the severity of influenza infections. Third, temporality has not been considered, and results are calculated on the basis of average annual influenza incidence. In addition, notified cases of influenza reported to the Department of Health may not reflect the true burden of the disease in the community because of inconsistencies in reporting. The hidden burden of infection maybe attributable to the increasing use of RATs, the results from which are not captured, and may also result from low healthcare seeking behaviour, especially among working age groups. We also acknowledged that the study could not estimate asymptomatic infections of influenza in the community. Thus, future studies should incorporate influenza surveillance, as well as whole genome sequencing data to better identify geographical areas at increased risk of influenza infection. Future studies should also incorporate multi-year vaccination data to better understand the relationship between vaccination coverage and influenza incidence rates.

## Conclusion

We found spatial clustering of influenza incidence in WA, suggesting that interventions could be geographically targeted. We produced maps showing the spatial clustering of influenza incidence in WA using routinely collected surveillance data. These maps may help the WA Department of Health to design strategies and target interventions for increased prevention and control of influenza in high-risk areas. Further nationwide studies using genomic and survey data might be required to better understand why distance to health facilities was negatively associated with influenza incidence.

## Ethical approval

Ethics approval for this research was granted by the Department of Health WA Human Research Ethics Committee (PRN ref: RGS0000006139; Information and Systems Performance Directorate Client Services project number: 202305.07).

## Authors’ contributions

Conceptualization KAA, HCM and CCB; data curation KAA, RP, and BG; formal analysis KAA; funding acquisition KAA, HCM, CCB, RP and BS; investigation KAA, HCM, CCB, RP, and BS; methodology KAA; project administration RP; resources KAA, HCM,CCB, RP, and BS; software KAA; supervision KAA, HCM, CCB, RP and BS; validation, visualization and writing of the original draft KAA; writing-review and editing KAA, HCM, ACAC, BG, DDB, RP, BS and CCB.

KAA, HCM and CCB directly accessed and verified the underlying data reported in the manuscript. No authors were precluded from accessing the data in the study and all authors accept responsibility to submit findings for publication.

## Data sharing statement

Requests to access influenza case data will need to be made to the data custodian West Australian Notifiable Infectious Disease Database (WANIDD). Requests to access influenza vaccination data will need to be made to the Australian Immunization Registry. The data sources of all other covariates analysed, are publicly available.

## Funding

This study was funded by Wesfarmers Centre of Vaccines and Infectious Diseases. The funding source had no role in the study design, its execution, analyses, interpretation of data or the decision to publish.

## Declaration of competing interest

The authors declare that they have no known competing financial interests or personal relationships that could have appeared to influence the work reported in this paper.
