# Supplementary material for: Spatiotemporal patterns of influenza in Western Australia
Source: Public Health Pract (Oxf). 2025 Mar 15;9:100602. doi: 10.1016/j.puhip.2025.100602 (PMC11982041; doi:10.1016/j.puhip.2025.100602)
Supplement: Multimedia component 1 [file mmc1.pdf]

Supplementary information

Supplementary Tables

Table S1: Data sources and definitions of covariates

Covariates	Data sources	Definitions
Influenza cases	WA Notifiable Infectious Diseases Database (WANIDD)	Reported number of influenzas cases aggregated by week, postcode, and age group
Influenza vaccination coverage	WA Health Department	The proportion of people vaccinated against the influenza virus
Socio-economic index*	Australian Bureau of Statistics	Index of relative socio-economic decile for each postcode
Population data	Australian Bureau of Statistics	Number of populations at postcode level
Population density	Calculated using population data	Number of populations per square kilometres
Temperature	WorldClime	Annual mean environmental air temperature (°C) (20)
Precipitation	WorldClime	Annual mean rainfall (mm) (20)
Access to healthcare facilities	Malaria Atlas Project (MAP)	Walking travel times in minutes to the nearest health facility (21)
WA polygon shapefile	Global Administrative Areas (GADM)	WA's administrative boundaries at Statistical Areas Level 3

Socio-economic indices include index of relative socio-economic disadvantage decile, index of relative socio-economic advantage and disadvantage decile, index of economic resources decile, and index of education and occupation decile

Table S2: Number of cases without geographic locations, stratified by age and years (n=72)

Variables	Number	Percent	Ratio*
**Year**			
2017	17	23.6	0.0028
2018	16	22.2	0.0027
2019	34	47.2	0.0015
2020	5	6.9	0.0042
**Age group**			
<6m	3	4.2	0.0147
6m-4y	4	5.6	0.0002
5-14y	9	12.5	0.0012
15-64y	41	56.9	0.0065
65+y	15	20.8	0.0044

*Number of influenzas cases without geographic areas divided by number of influenza cases with geographic location.

Supplementary Figures

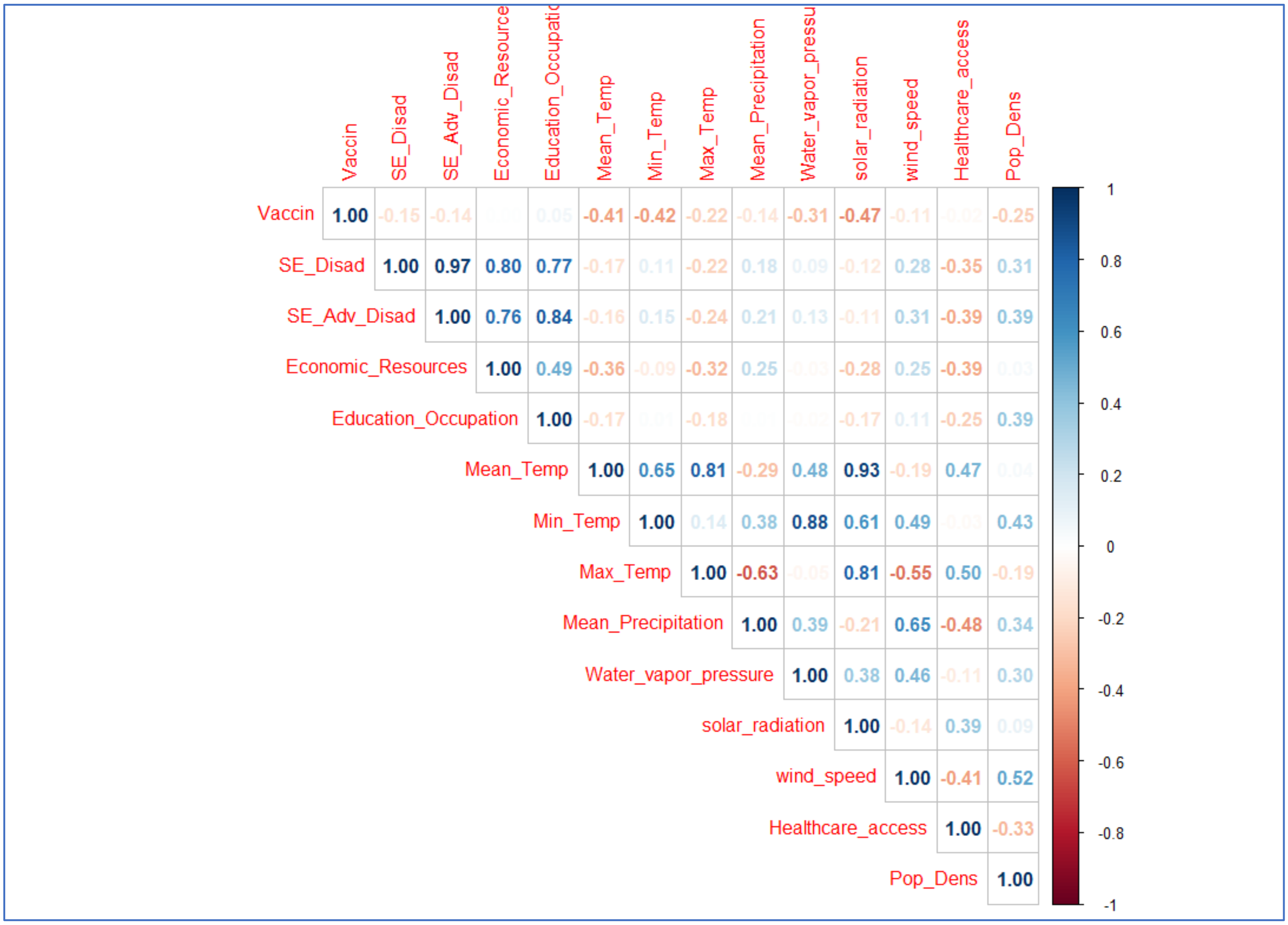

Figure S1: Pearson correlations between all covariates**.**

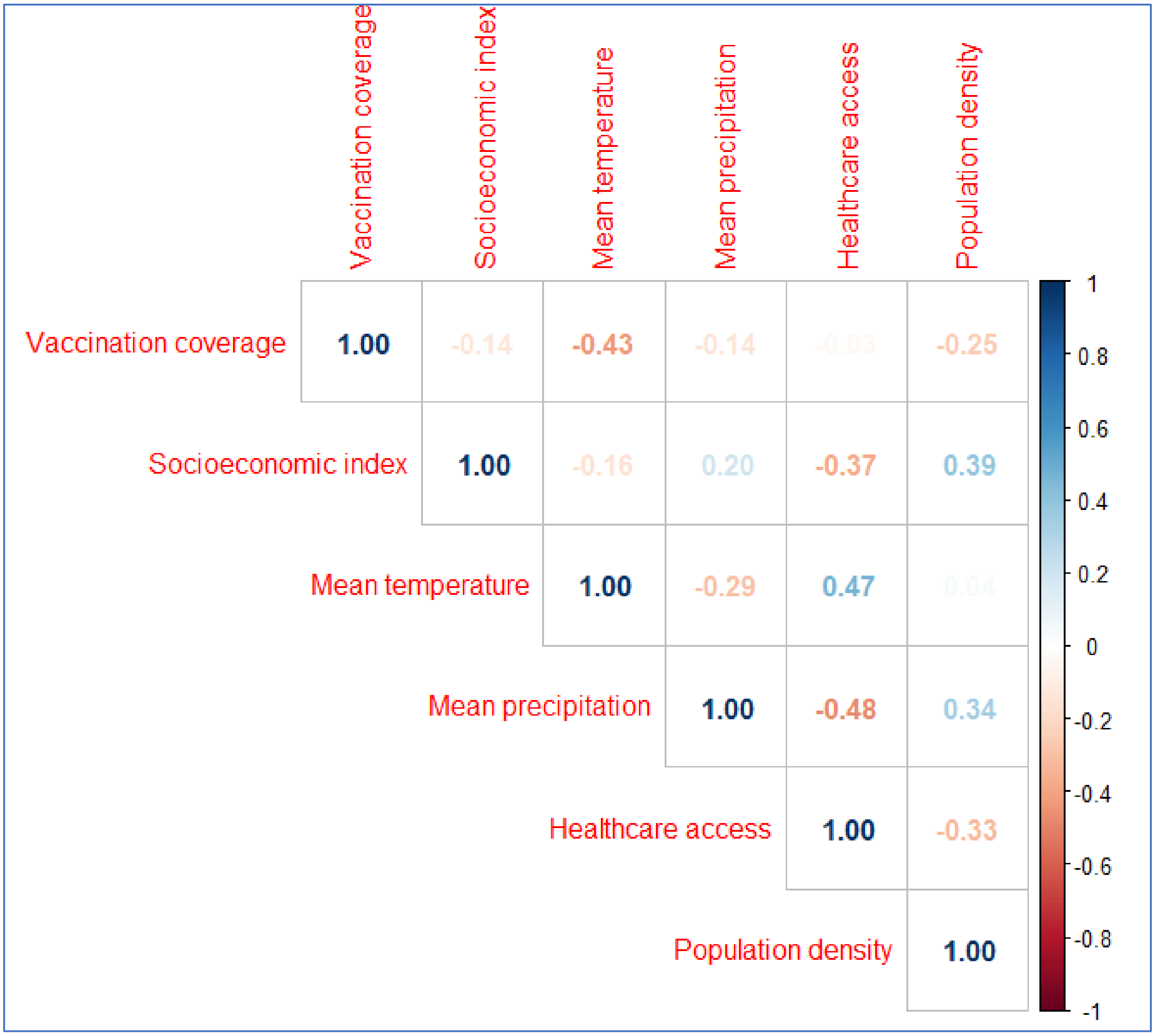

Figure S2: Pearson correlations between covariates included in the model

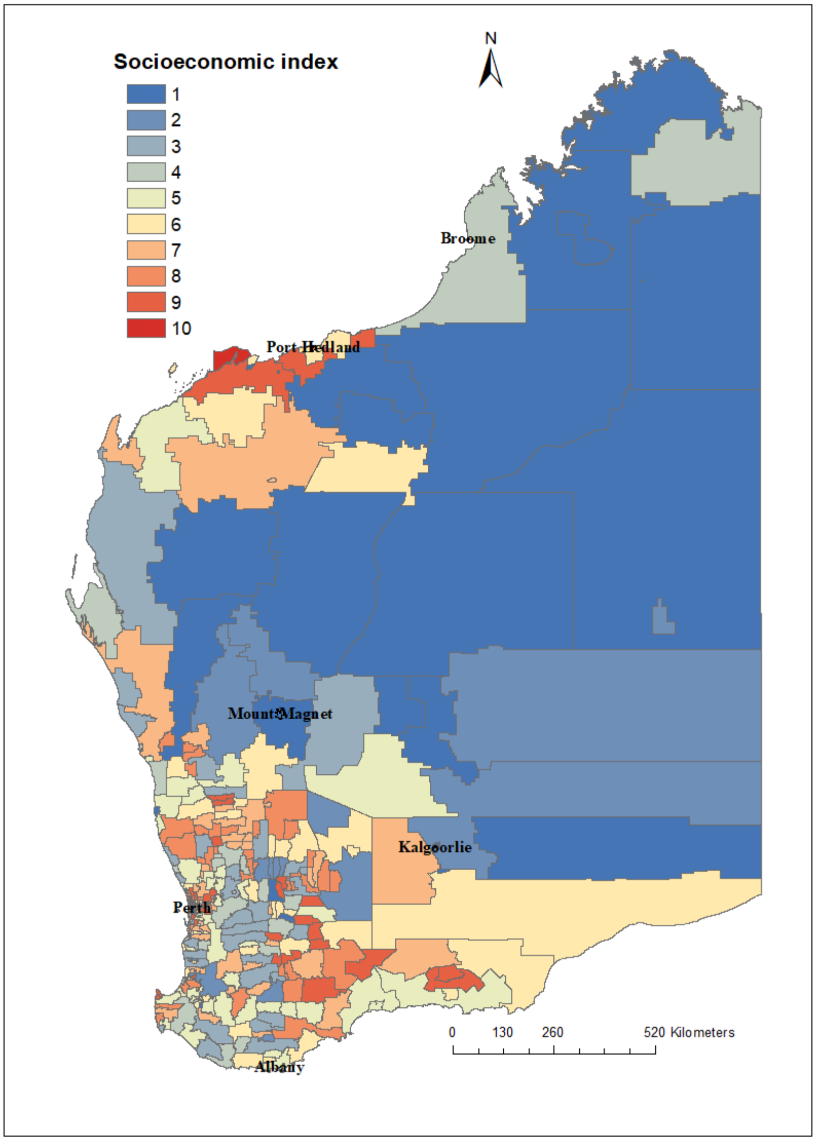

Figure S3: Socioeconomic index (i.e., index of relative socio-economic advantage and disadvantage) for Western Australia, 2016.

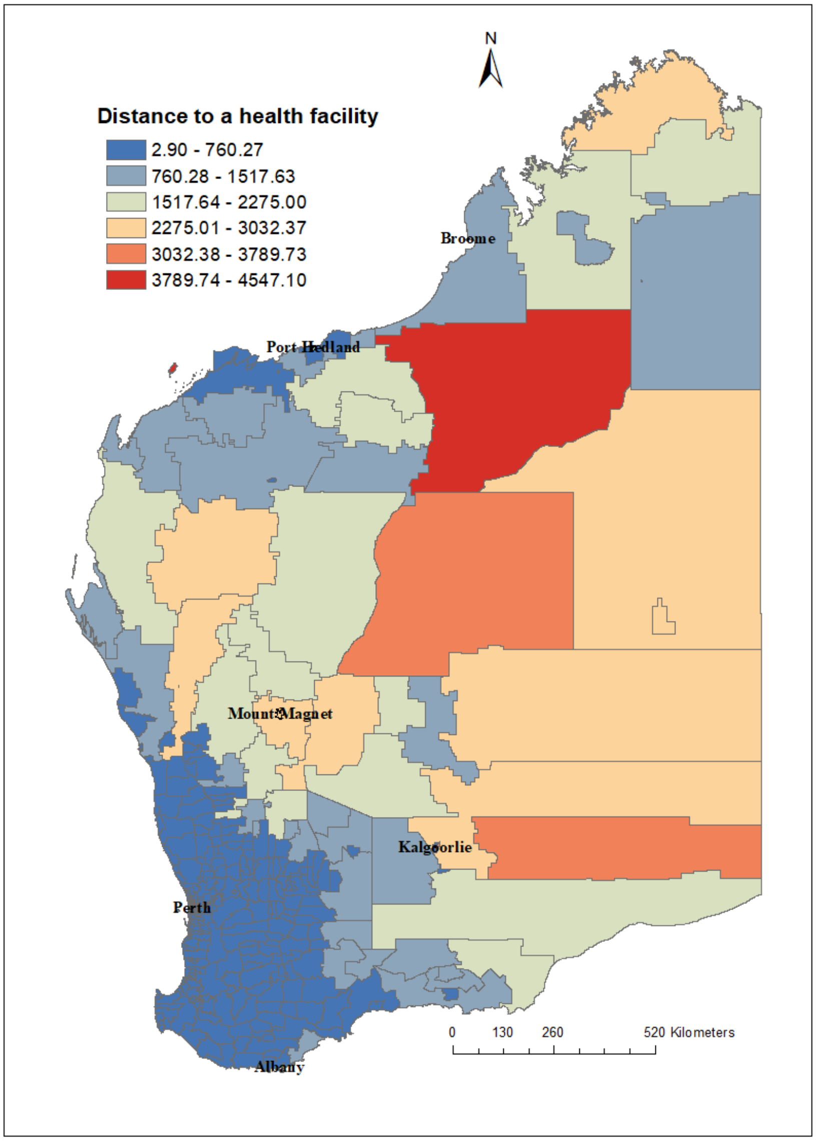

Figure S4: Walking distance to the nearest health facilities in minutes in Western Australia, 2020.

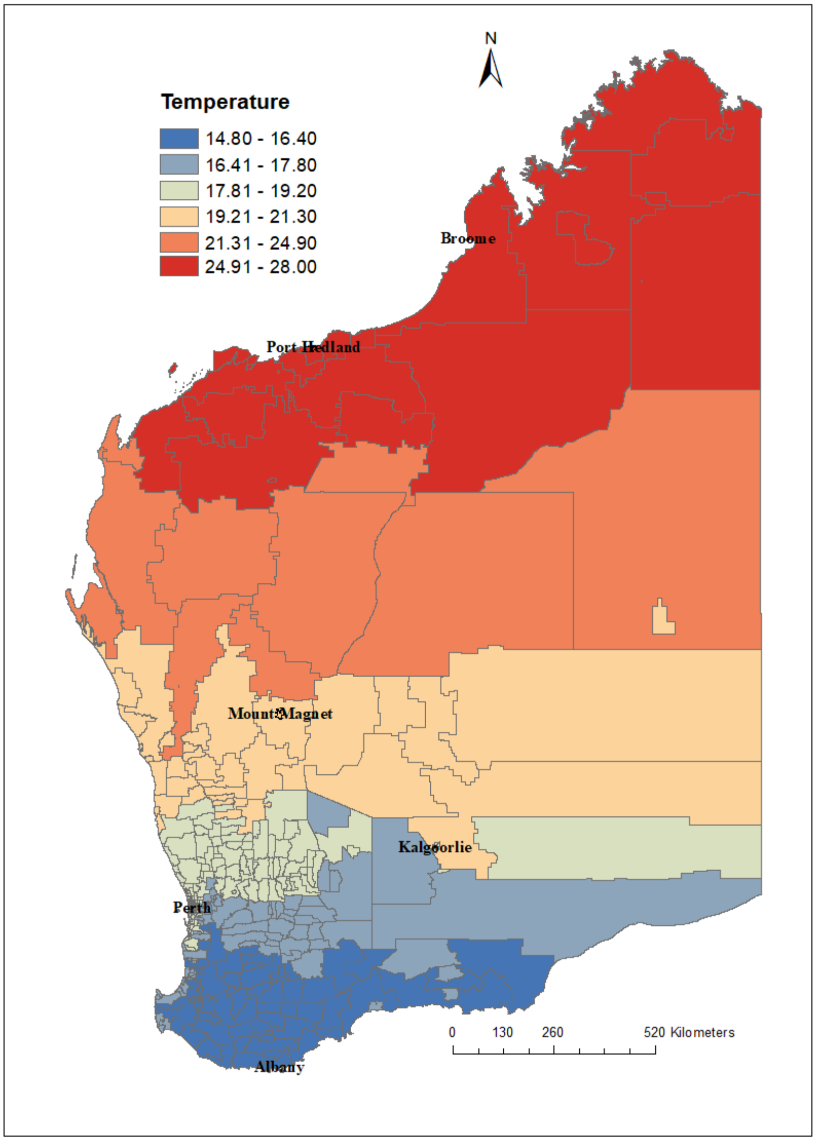

Figure S5: Annual mean temperature in degree Celsius at postcode level in WA, 2017-2020

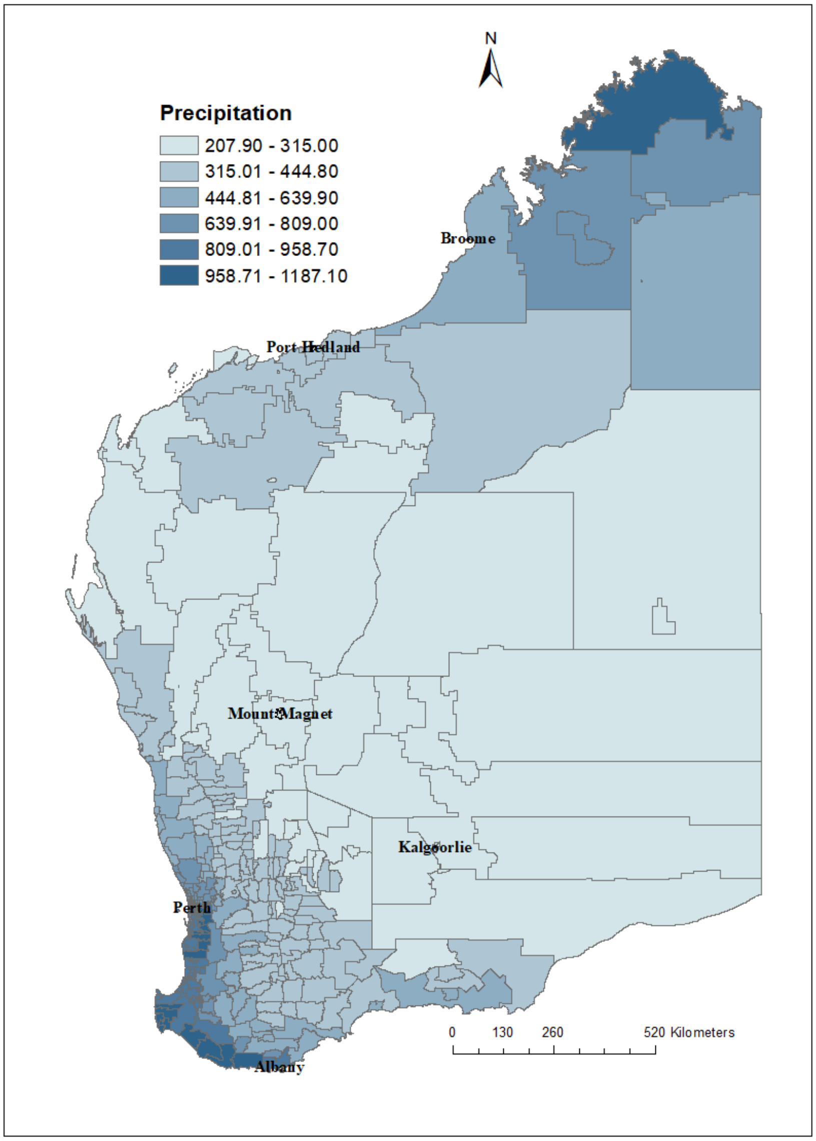

Figure S6: Annual mean precipitation (mm) at postcode level in Western Australia, 2017-2020

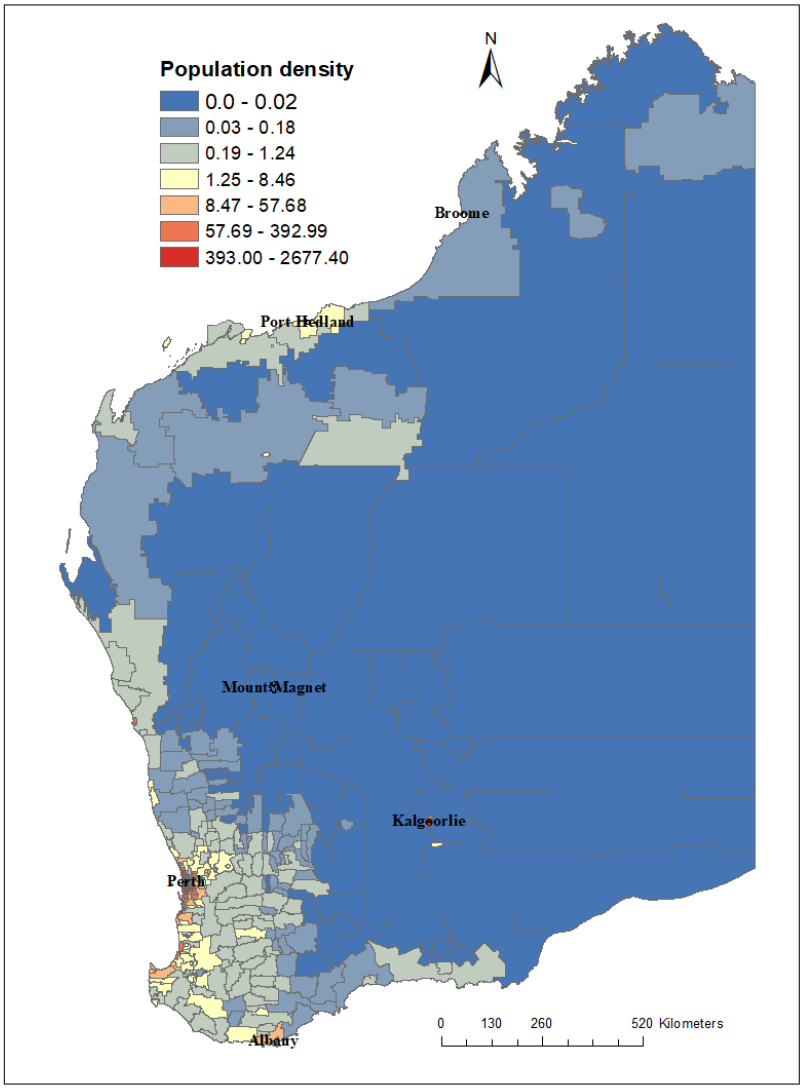

Figure S7: Population density (i.e., number of persons per square kilometre) in Western Australia, 2017-2020

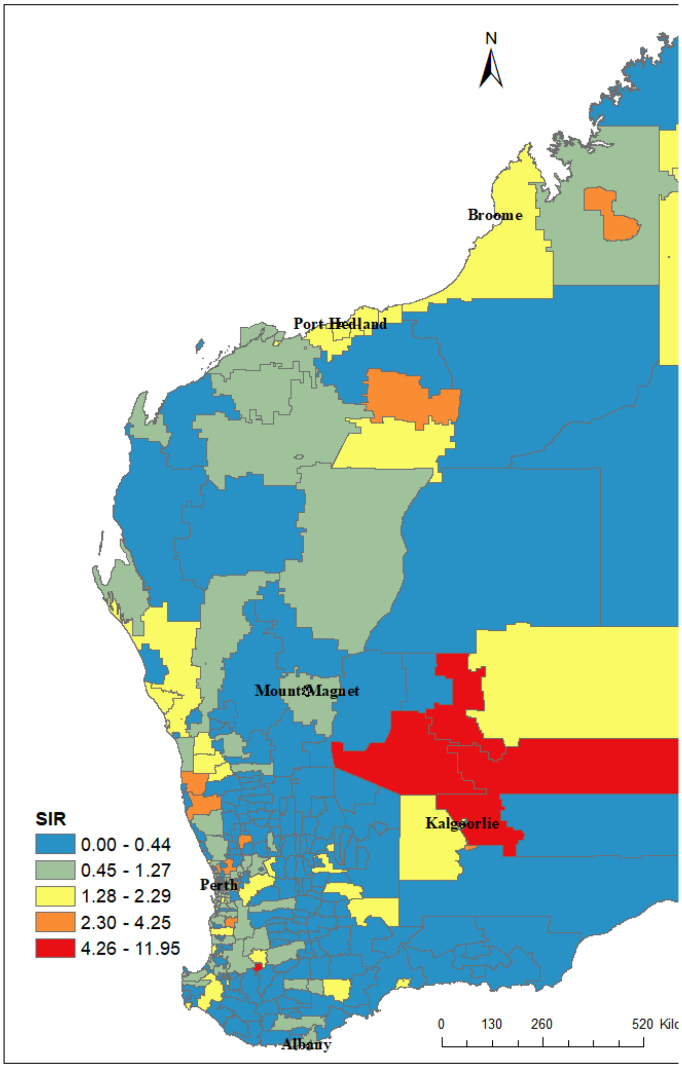

Figure S8a: SIR with age less than 5 years

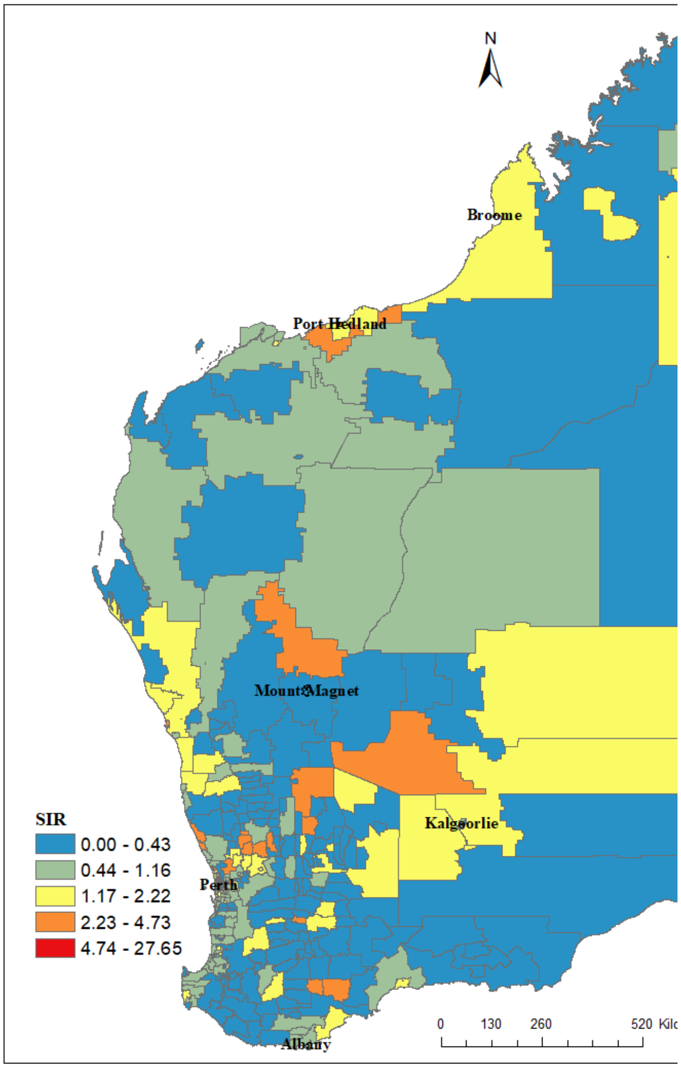

Figure S8b: SIR with age between 5 and 14 years

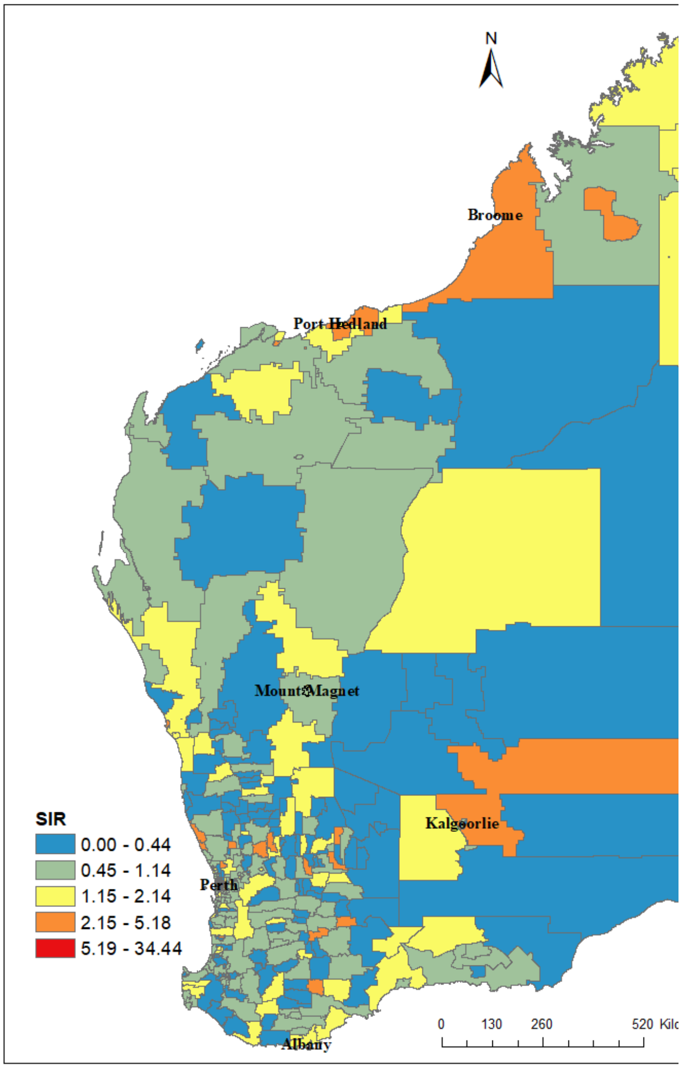

Figure S8c: SIR with age between 15-64 years

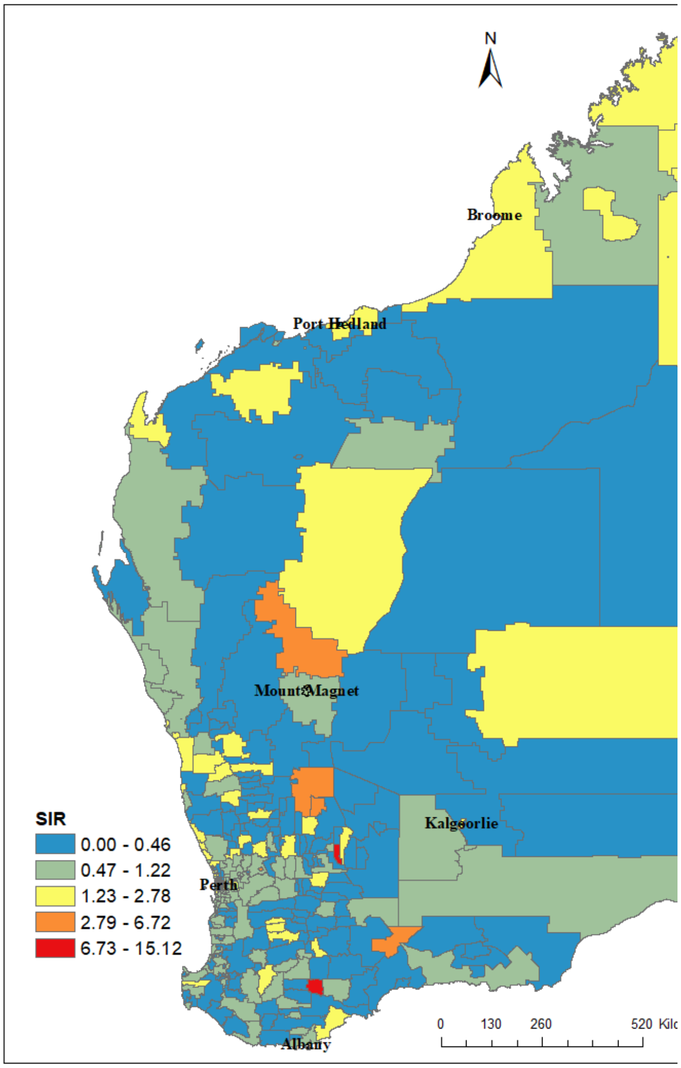

Figure S8d: SIR with age 65 years and above

Figure S8: Year standardized incidence rate (SIR) of influenza by age group at postcode level in Perth areas, 2017–2020.

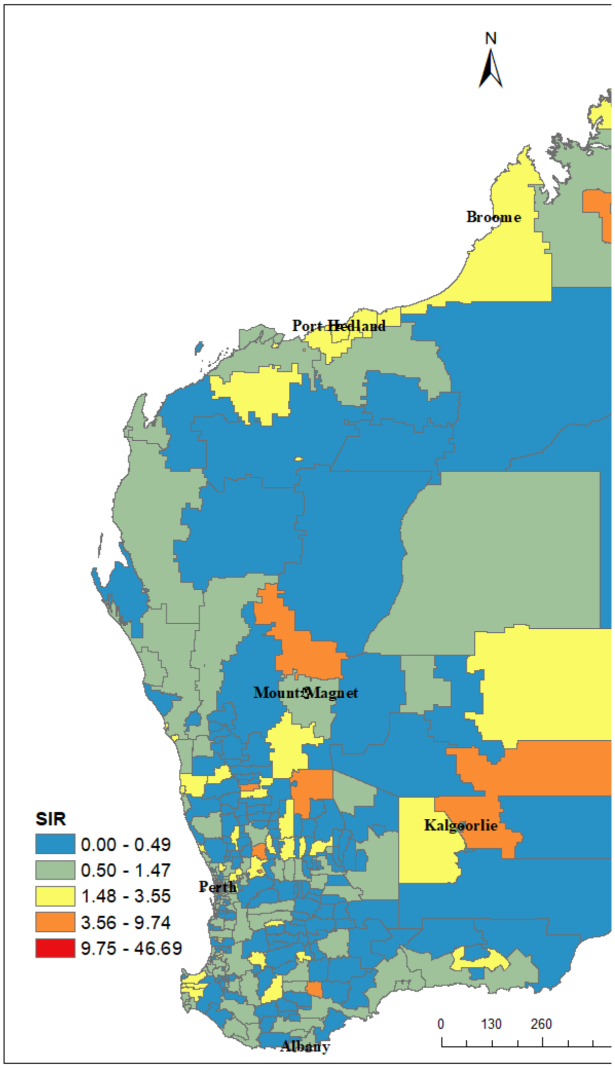

Figure S9a: SIR in 2017

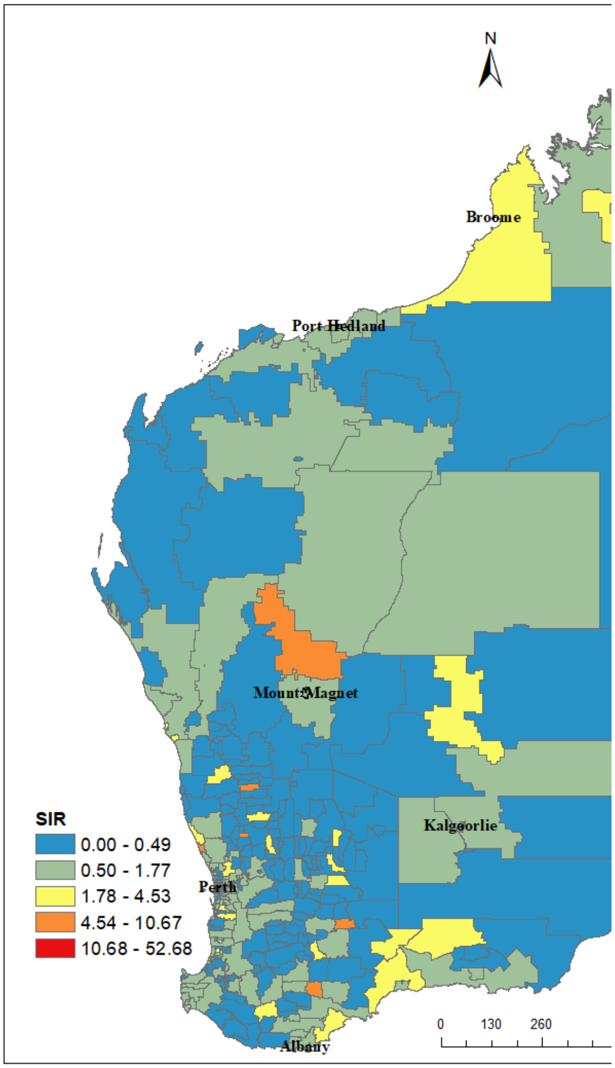

Figure S9b: SIR in 2018

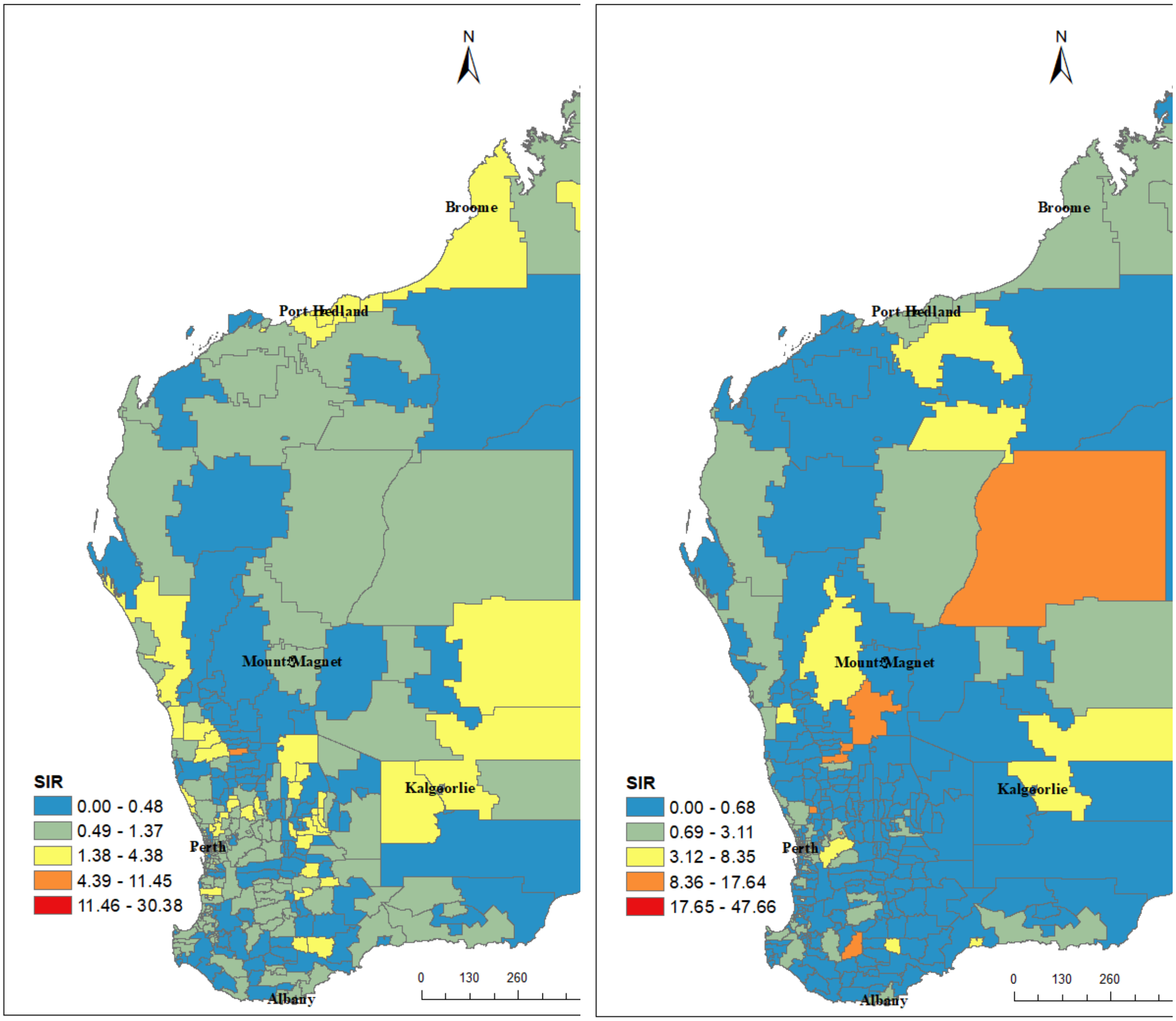

Figure S9c: SIR in 2019 Figure S9d: SIR in 2020

Figure S9: Age standardized incidence rate (SIR) of influenza by year at postcode level in Western Australia.

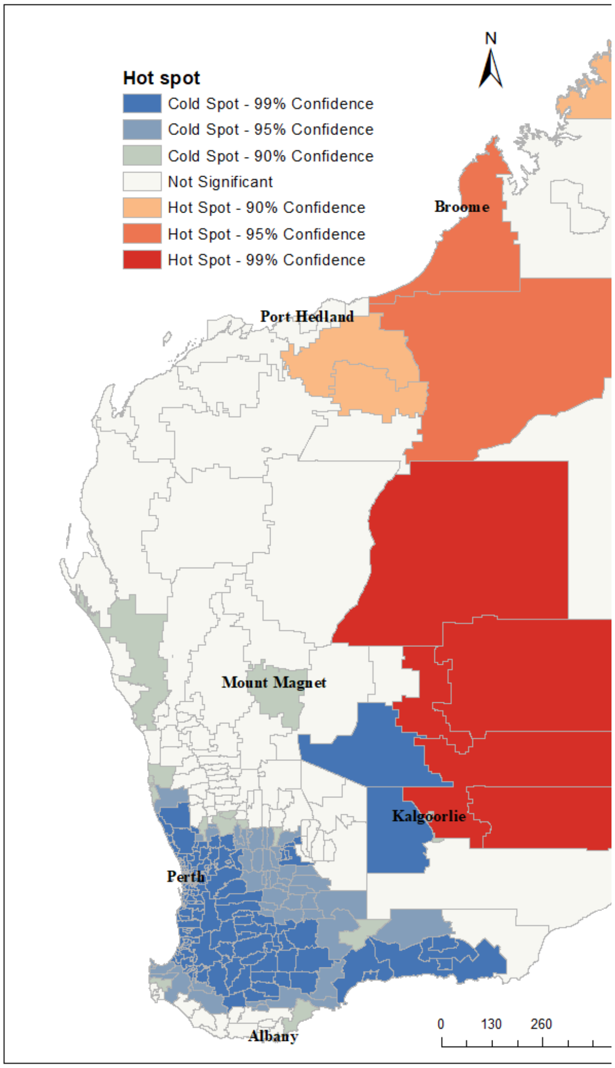

Figure S10a: Spatial clustering in age less than 5 years

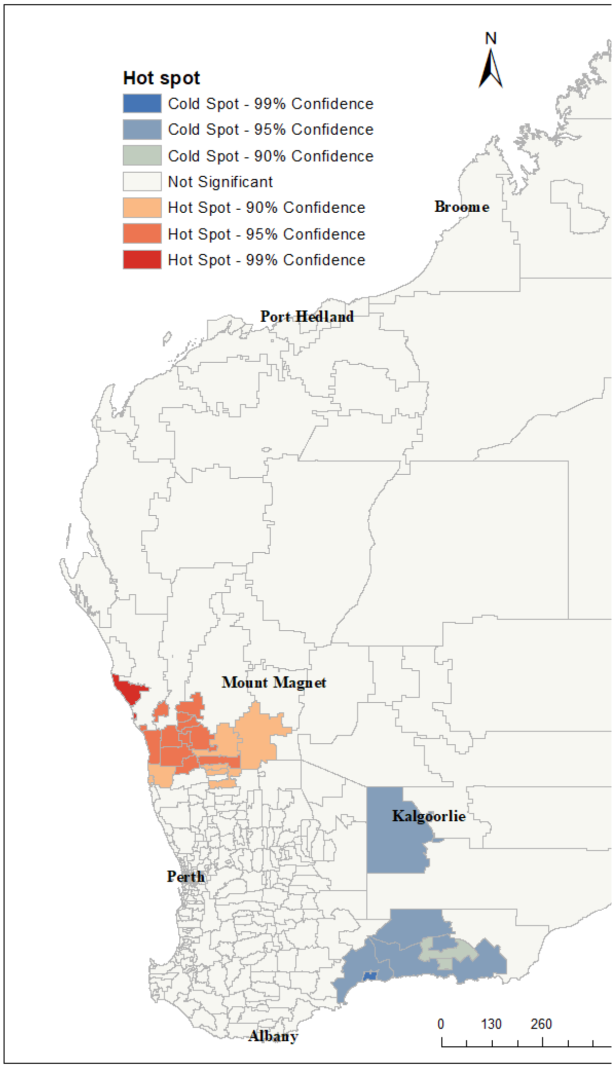

Figure S10b: Spatial clustering in age between 5 -14 years

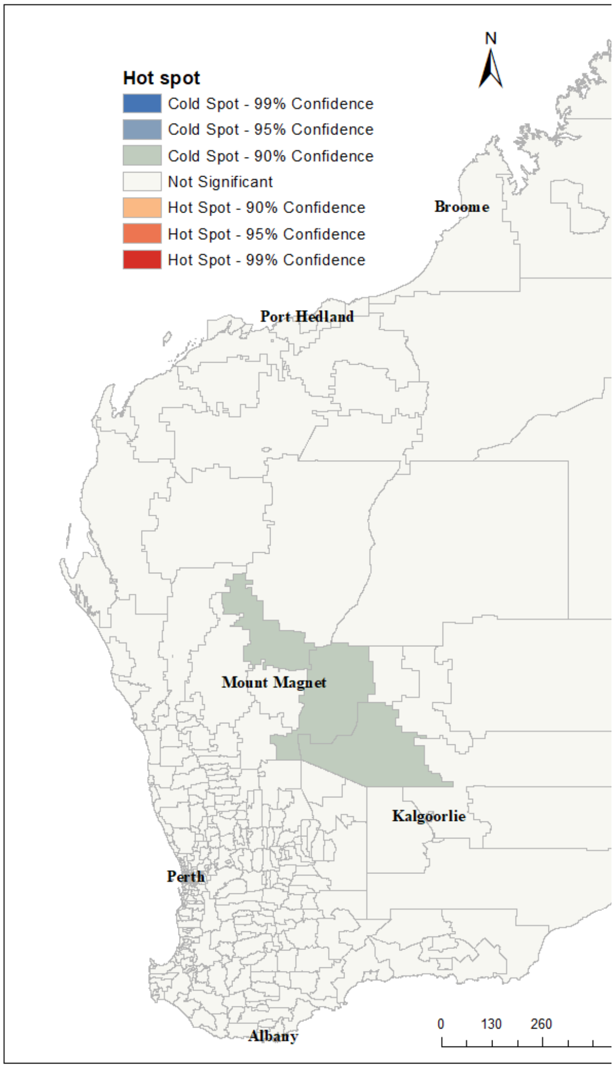

Figure S10c: Spatial clustering in age between 15-64 years

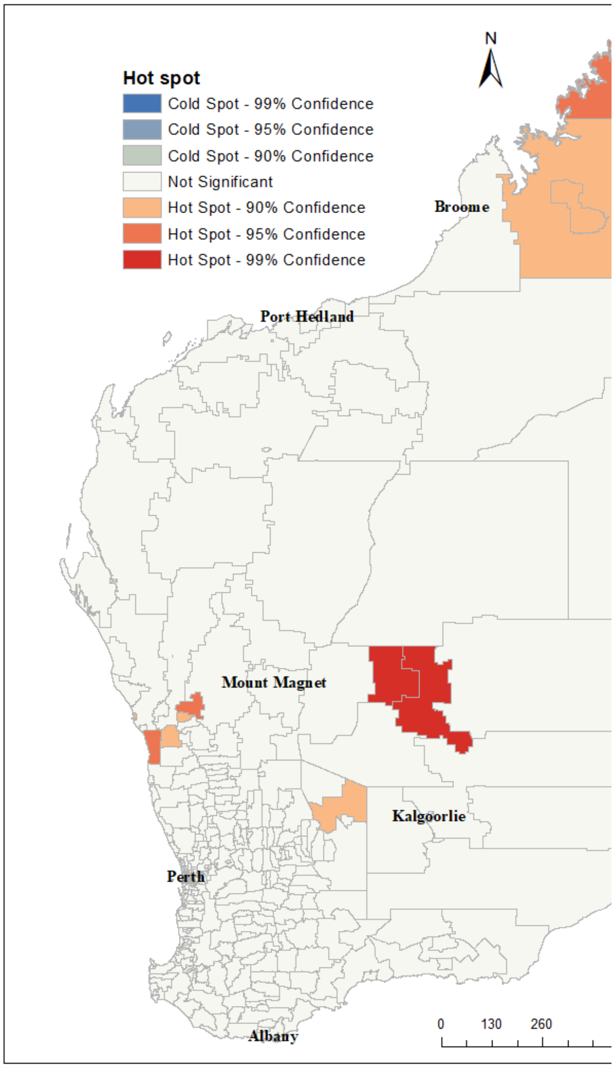

Figure S10d: Spatial clustering with age 65 years and above

Figure S10: Spatial clustering of influenza incidence by age group in Western Australia based on the Getis-Ord Gi* statistics, 2017-2020.

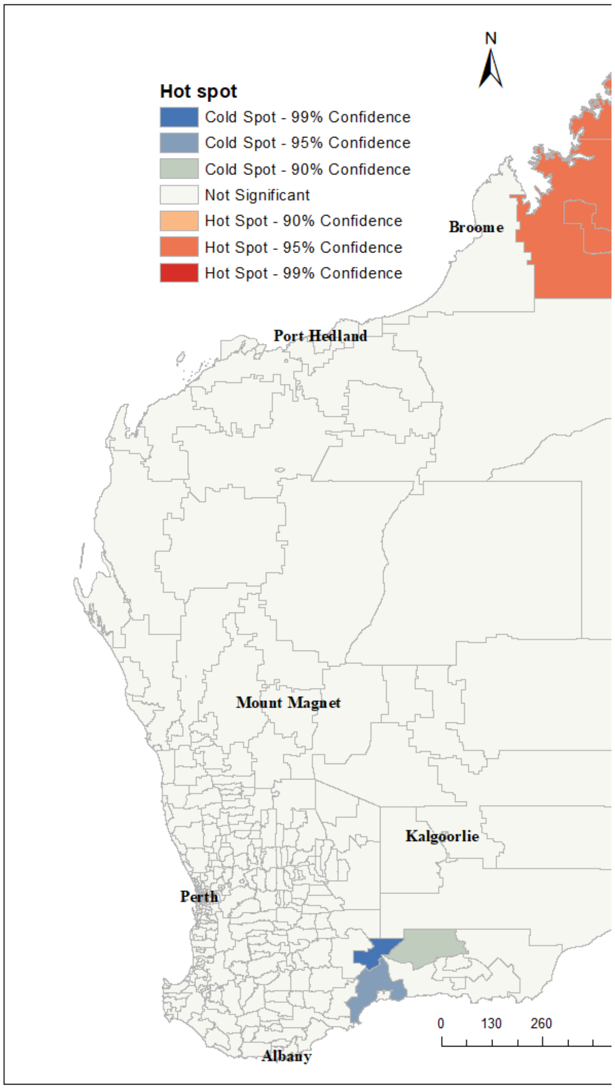

Figure S11a: Spatial clustering in 2017

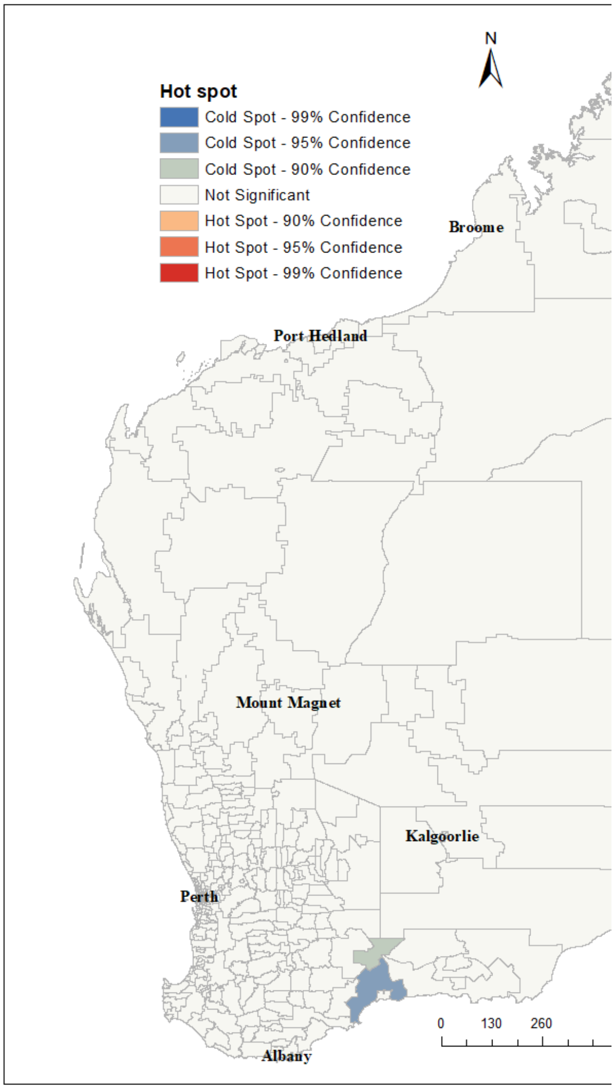

Figure S11b: Spatial clustering in 2018

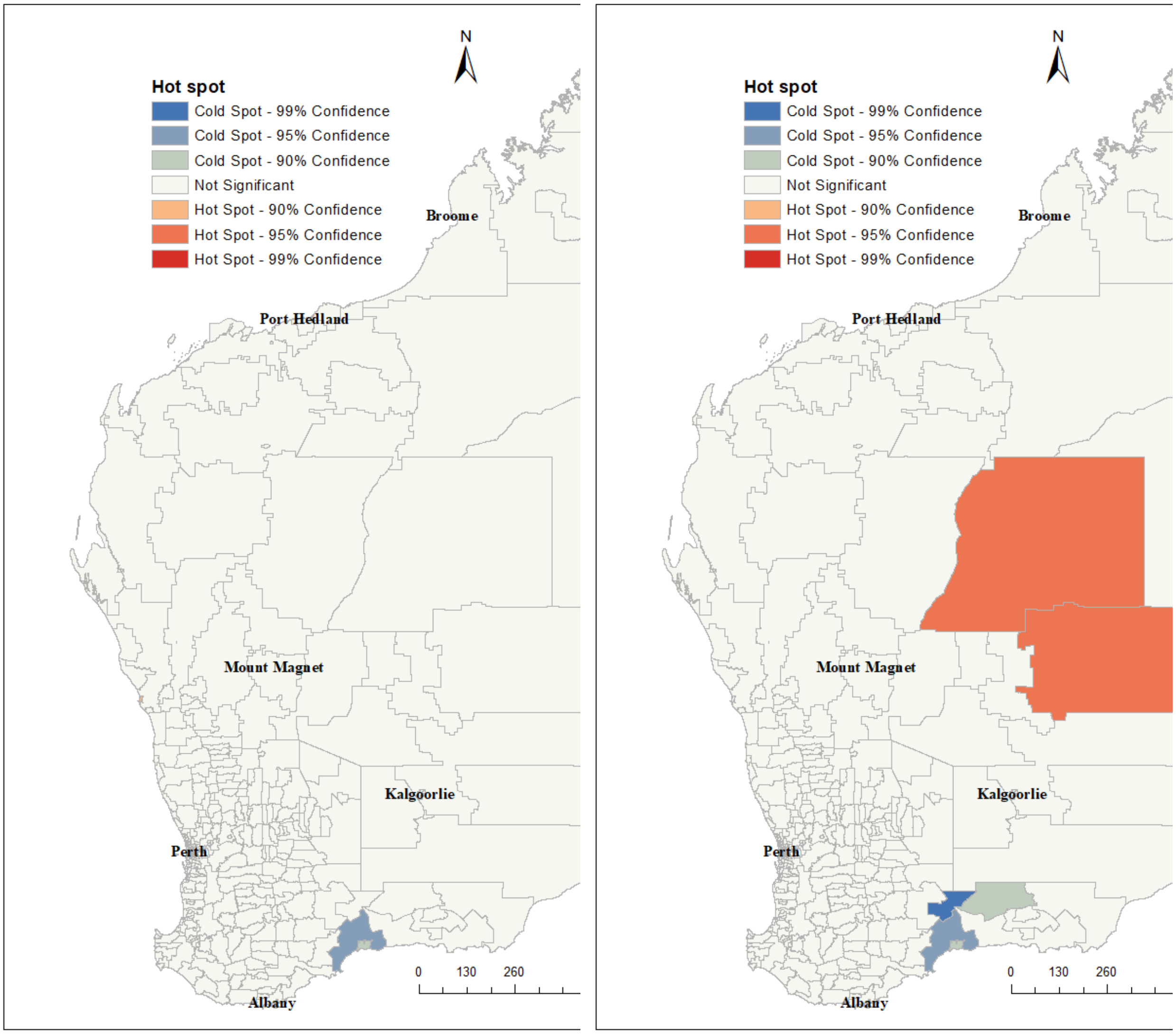

Figure S11c: Spatial clustering in 2019   Figure S11d: Spatial clustering in 2020

Figure S11: Spatial clustering of influenza incidence in Western Australia by year based on the Getis-Ord Gi* statistics.

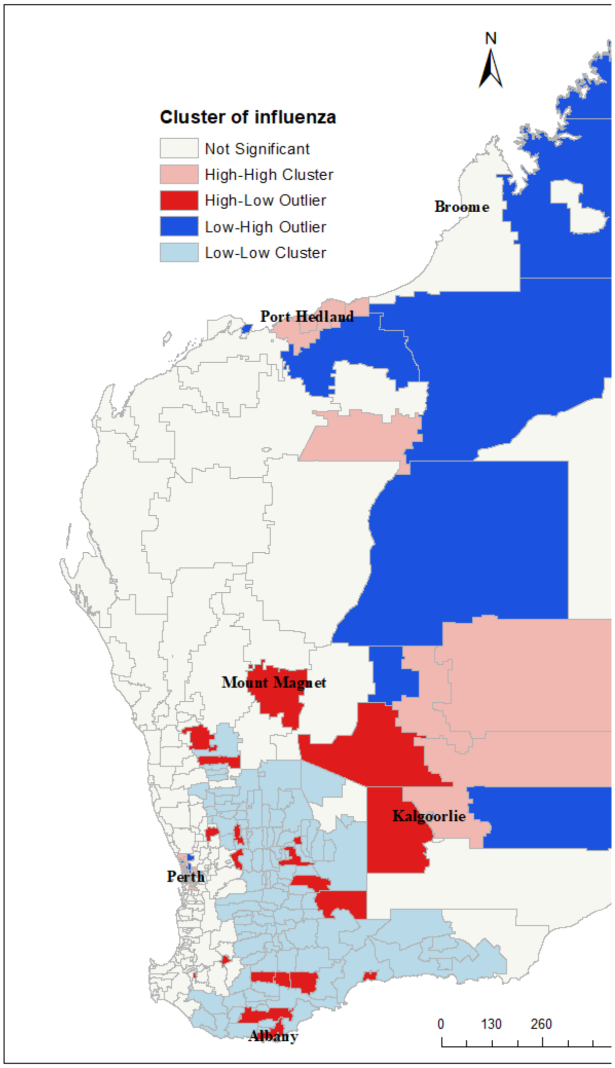

Figure S12a: Spatial clustering in age less than 5 years

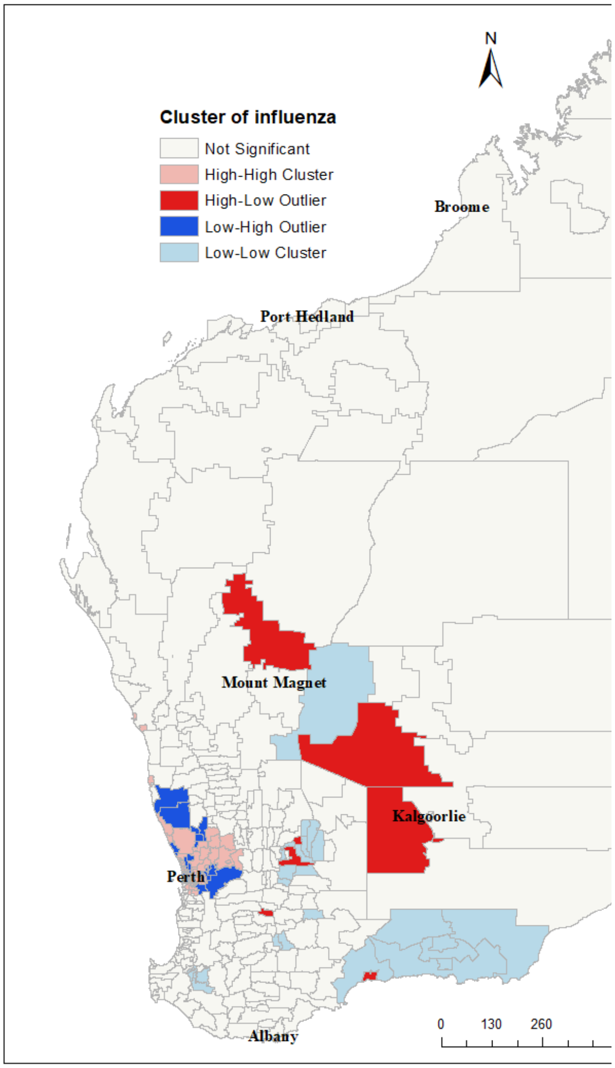

Figure S12b: Spatial clustering in age between 5 -14 years

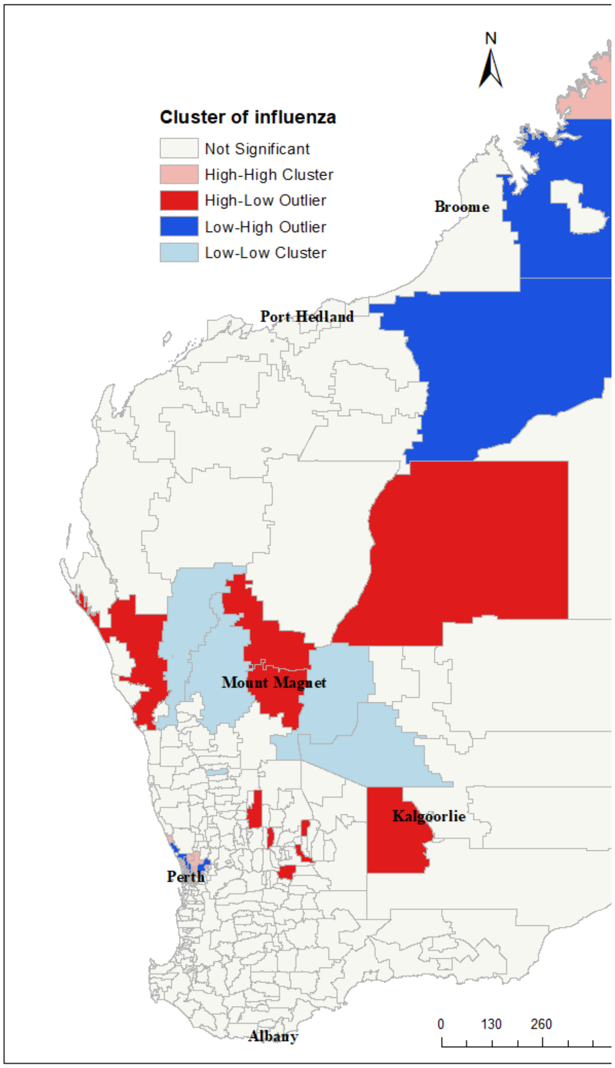

Figure S12c: Spatial clustering in age between 15-64 years

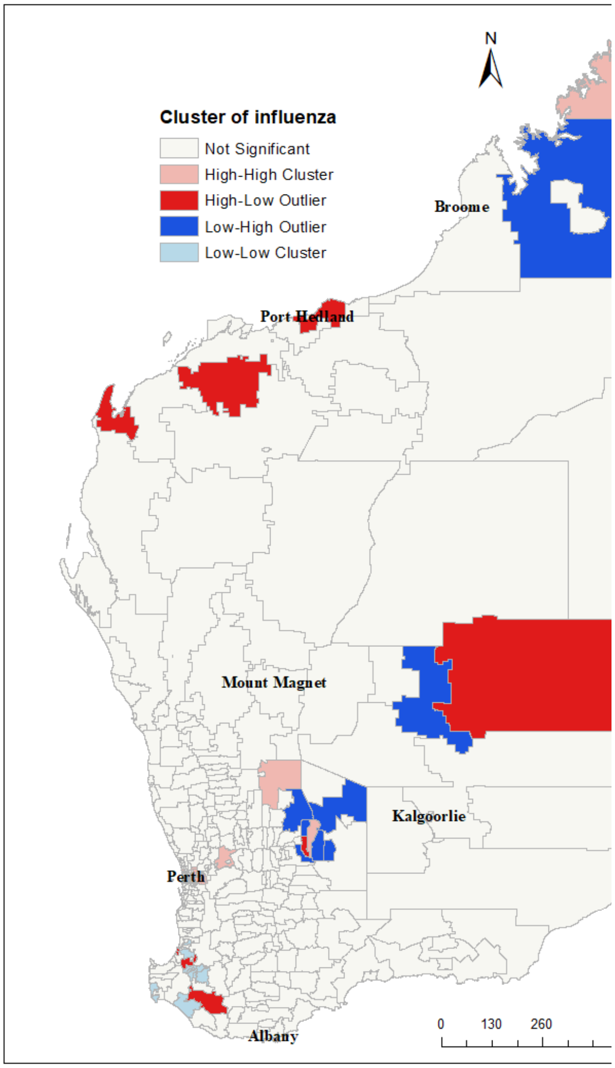

Figure S12d: Spatial clustering with age 65 years and above

Figure S12: Spatial clustering of influenza incidence in Western Australia by age group based on local Moran's I statistics, 2017-2020.

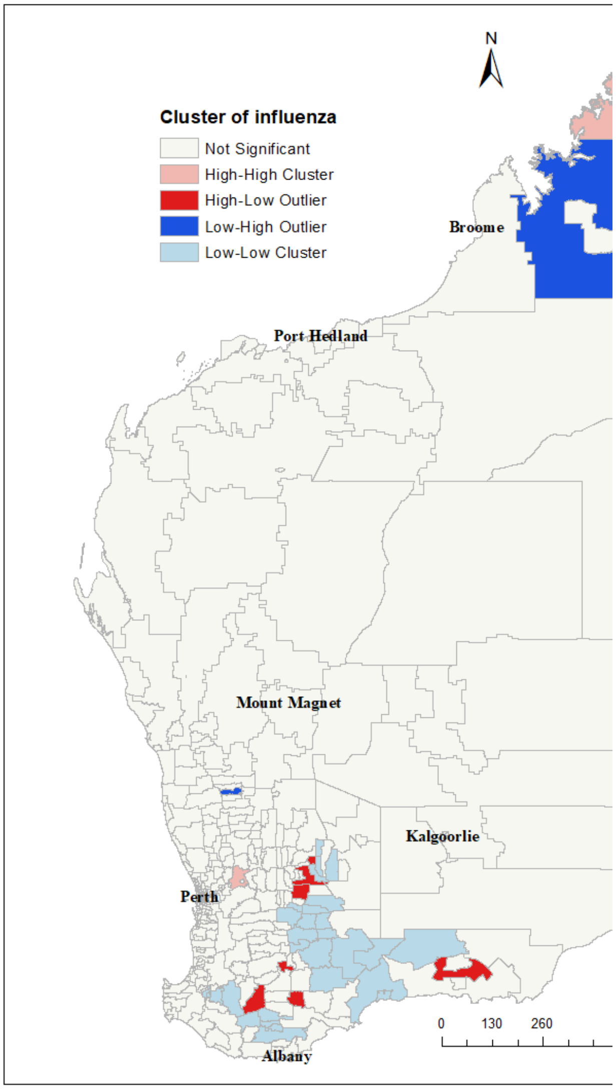

Figure S13a: Spatial clustering in 2017

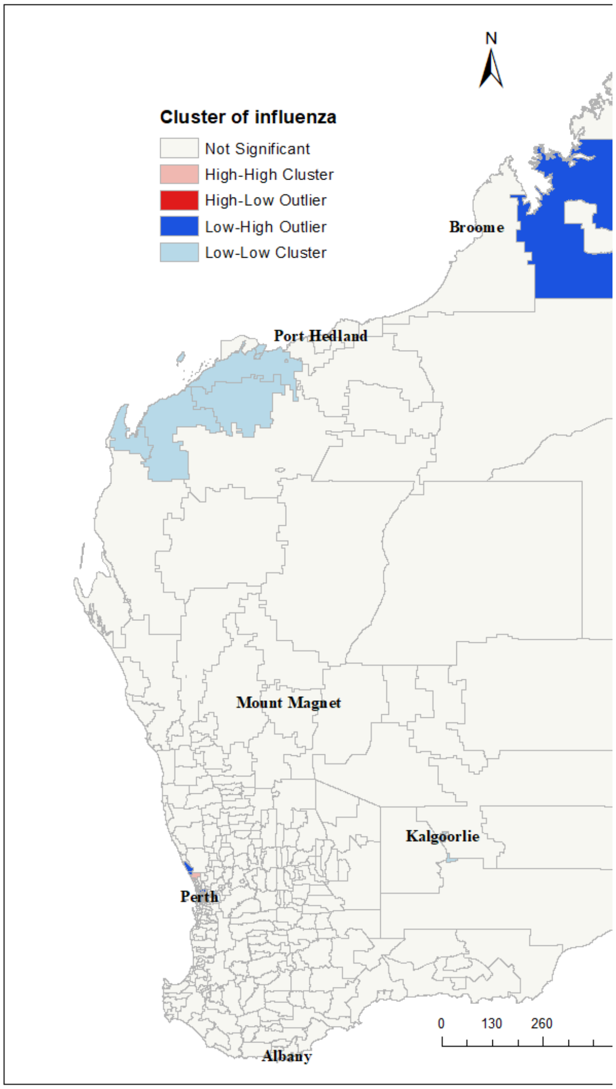

Figure S13b: Spatial clustering in 2018

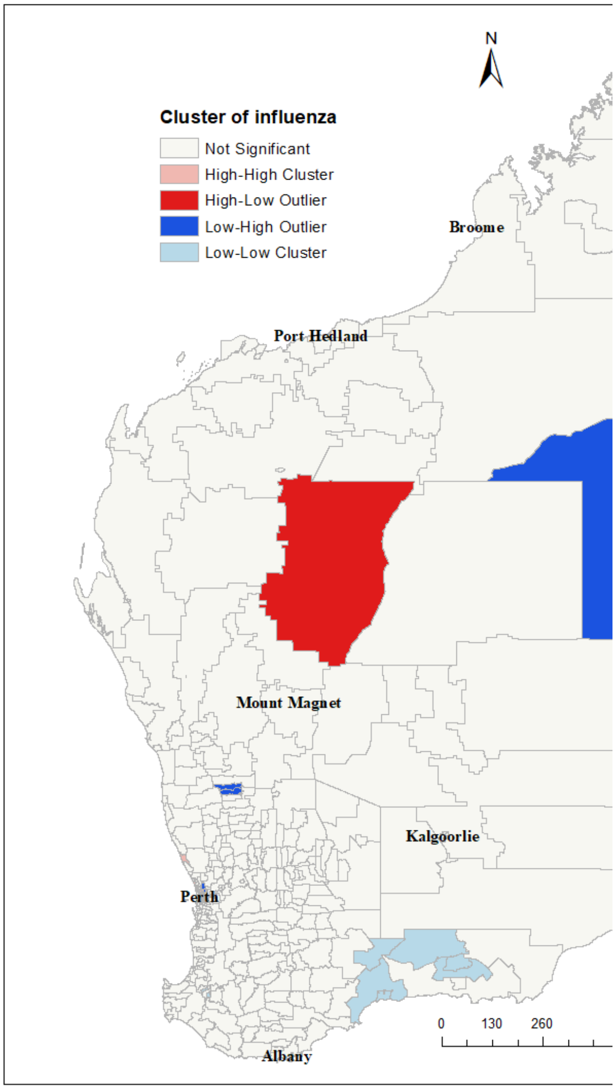

Figure S13c: Spatial clustering in 2019

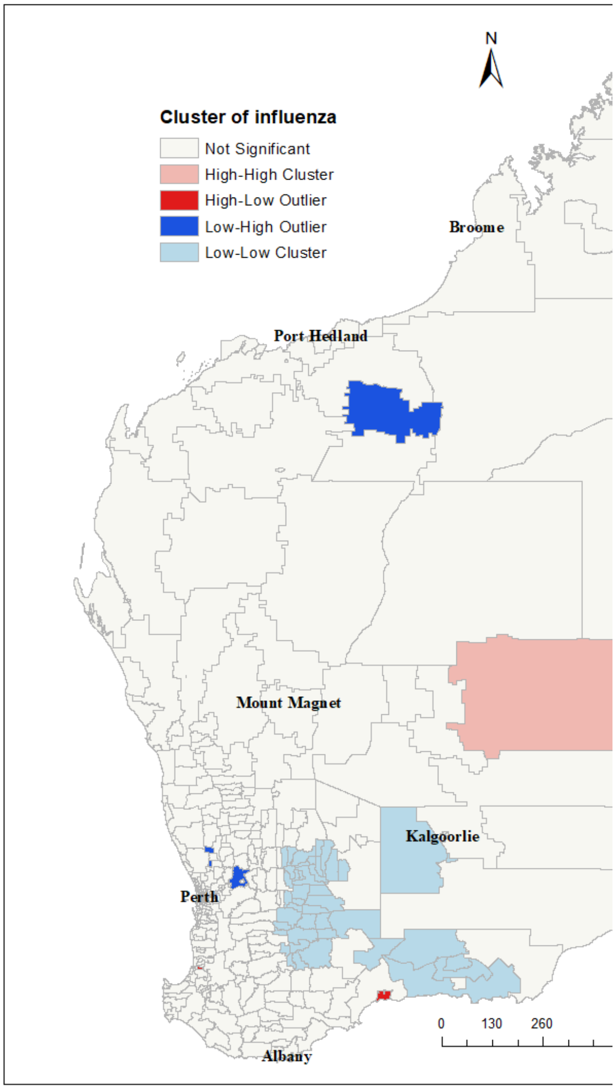

Figure S13d: Spatial clustering in 2020

Figure S13: Spatial clustering of influenza incidence in Western Australia by year based on local Moran's I statistics.
